# Supplementary material for: Nurses’ 12-hour shifts and missed or delayed vital signs observations on hospital wards: retrospective observational study
Source: BMJ Open. 2019 Feb 1;9(1):e024778. doi: 10.1136/bmjopen-2018-024778 (PMC6361343; doi:10.1136/bmjopen-2018-024778)
Supplement: Supplementary data [file bmjopen-2018-024778supp001.pdf]

Appendix 1 – HPPD, number of nursing staff and number of beds by ward

Ward	Number of beds	Number of Nursing Staff	HPPD Mean (SD)	Skill Mix* Mean (SD)
Oncology	40	109	7.2 (0.8)	0.71 (0.02)
Medical – Gastro	36	72	6.4 (1)	0.50 (0.07)
Coronary Care	23	48	7.2 (0.8)	0.83 (0.06)
Cardiology	36	62	5.6 (0.7)	0.51 (0.05)
Trauma 1	28	59	7.3 (1.1)	0.54 (0.06)
General Medicine 1	30	47	6.5 (1)	0.45 (0.05)
General Medicine 2	34	58	5.5 (0.8)	0.58 (0.05)
Trauma 2	26	55	6.8 (0.8)	0.55 (0.05)
Orthopaedics 1	36	46	6.4 (1.2)	0.56 (0.05)
Orthopaedics 2	36	62	7.4 (1.2)	0.52 (0.05)
Orthopaedics 3	13	31	8.1 (1.3)	0.56 (0.06)
Surgical 1	30	56	5.2 (0.5)	0.60 (0.05)
Surgical 2	32	61	5.4 (0.7)	0.59 (0.05)
Respiratory 1	36	60	5.4 (0.8)	0.50 (0.07)
Respiratory 2	40	83	7.1 (0.6)	0.67 (0.04)
Medicine for older people 1	13	79	7.7 (1.4)	0.50 (0.06)
Medicine for older people 2	30	25	7.3 (0.9)	0.46 (0.05)
Medicine for older people 3	23	73	6.5 (0.6)	0.52 (0.04)
Medicine for older people 4	29	84	6.9 (0.8)	0.52 (0.05)
Medicine for older people 5	30	49	7.6 (0.9)	0.47 (0.08)
Stroke Unit 1	25	62	7 (0.5)	0.46 (0.05)
Stroke Unit 2	34	85	6.7 (0.8)	0.53 (0.06)
Renal 1	10	47	11.2 (1.6)	0.78 (0.06)
Renal 2	26	56	8.4 (1)	0.79 (0.05)
Renal 3	14	31	8.6 (1.2)	0.75 (0.05)
Gynaecology	22	46	7.9 (1.5)	0.66 (0.07)
Head & Neck	27	51	6.3 (1.2)	0.68 (0.07)
Medical Assessment Unit	58	155	8.9 (1.3)	0.71 (0.03)
Private Patients Unit	13	30	8.3 (1.8)	0.69 (0.05)
Surgical Assessment Unit	28	53	5.2 (0.9)	0.65 (0.06)
Surgical High Intensity Unit	10	31	10 (1.5)	0.79 (0.06)
Urology	31	78	5.6 (0.7)	0.55 (0.04)

* Defined as RN-HPPD/(RN-HPPD + HCA-HPPD)
