# Supplementary material for: Nurses’ 12-hour shifts and missed or delayed vital signs observations on hospital wards: retrospective observational study
Source: BMJ Open. 2019 Feb 1;9(1):e024778. doi: 10.1136/bmjopen-2018-024778 (PMC6361343; doi:10.1136/bmjopen-2018-024778)
Supplement: Author's manuscript [file bmjopen-2018-024778.draft_revisions.pdf]

BMJ Open

BMJ Open is committed to open peer review. As part of this commitment we make the peer review history of every article we publish publicly available.

When an article is published we post the peer reviewers' comments and the authors' responses online. We also post the versions of the paper that were used during peer review. These are the versions that the peer review comments apply to.

The versions of the paper that follow are the versions that were submitted during the peer review process. They are not the versions of record or the final published versions. They should not be cited or distributed as the published version of this manuscript.

BMJ Open is an open access journal and the full, final, typeset and author-corrected version of record of the manuscript is available on our site with no access controls, subscription charges or pay-per-view fees (http://bmjopen.bmj.com).

If you have any questions on BMJ Open's open peer review process please email info.bmjopen@bmj.com

BMJ Open

Nurses' 12-hour shifts and missed or delayed vital signs observations on hospital wards: retrospective observational study

Journal:	*BMJ Open*
Manuscript ID	bmjopen-2018-024778
Article Type:	Research
Date Submitted by the Author:	13-Jun-2018

Complete List of Authors:	Dall'Ora, Chiara; University of Southampton, Faculty of Health Sciences; NIHR Collaboration for Leadership in Applied Health Research and Care Wessex,<br>Griffiths, Peter; University of Southampton, Faculty of Health Sciences<br>Redfern, Oliver; University of Portsmouth, Centre for Healthcare Modelling and Informatics<br>Recio-Saucedo, Alejandra; University of Southampton, Faculty of Health Sciences<br>Meredith, Paul; Portsmouth Hospitals NHS Trust, Research & Innovation Department<br>Ball, Jane; National Institute for Health Research Collaboration for Leadership in Applied Health Research and Care (Wessex),University of Southampton, Centre for Innovation and Leadership in Health Sciences

Keywords:	Shift Work Schedule, Nursing, 12-hour Shifts, Vital Signs Monitoring, Missed Care

SCHOLARONE™
Manuscripts

TITLE PAGE

Nurses' 12-hour shifts and missed or delayed vital signs observations on hospital wards: retrospective observational study

Chiara Dall'Ora* [1,2] RN, MSc, PhD, University of Southampton, Building 67, Highfield, Southampton, SO17 1BJ, UK, C.Dall'ora@soton.ac.uk, +44(023) 8059 8249

Peter Griffiths*[1,2], Oliver Redfern[3], Alejandra Recio-Saucedo[2], Paul Meredith[4], Jane Ball[2], On behalf of the Missed Care Study Group[†]

* Chiara Dall'Ora and Peter Griffiths contributed equally to this manuscript

[1] National Institute for Health Research Collaboration for Leadership in Applied Health Research and Care (Wessex), University of Southampton, Southampton, UK

[2] Faculty of Health Sciences, University of Southampton, Southampton, UK

[3] School of Computing, University of Portsmouth, UK

[4] Research & Innovation Department, Portsmouth Hospitals NHS Trust, Portsmouth, UK

[†] The Missed Care Study Group comprises Karen Bloor, University of York; Dankmar Böhning, University of Southampton; Jim Briggs, University of Portsmouth, Centre for Healthcare Modelling and Informatics; Anya De Iongh, Independent lay researcher; Jeremy Jones, University of Southampton, Faculty of Health Sciences; Caroline Kovacs, University of Portsmouth, Centre for Healthcare Modelling and Informatics; David Prytherch University of Portsmouth, Centre for Healthcare Modelling and Informatics and Portsmouth Hospitals NHS Trust, Clinical Outcomes Research Group; Paul Schmidt, National Institute for Health Research Collaboration for Applied Health Research and Care (Wessex) and Portsmouth Hospitals NHS Trust, Clinical Outcomes Research Group; Nicky Sinden, Portsmouth Hospitals NHS Trust, Clinical Outcomes Research Group; Gary Smith, Bournemouth University

For peer review only

For peer review only - http://bmjopen.bmj.com/site/about/guidelines.xhtml

Word count: 2267

For peer review only

For peer review only - http://bmjopen.bmj.com/site/about/guidelines.xhtml

ABSTRACT

Objectives: 12-hour shifts worked by nurses on acute hospital wards have been associated with increased rates of missed care reported by nurses. This study aimed to measure the association between nurses working shifts of at least 12 hours and an objective measure of missed care – vital signs observations taken on time according to an acuity-based surveillance protocol.

Design: A retrospective observational study using routinely collected data from March 2012 to March 2015.

Setting: 32 general inpatient wards at a large acute hospital in England.

Participants: 658,628 nursing shifts nested in 24,069 ward-days.

Outcome measures: The rate of daily delayed and missed vital signs observations. We focussed on situations where vital signs observations were required at least every 4 hours and measured the number of instances where observations were delayed or missed, per 24-hour period. For each ward and each day, shift patterns were characterised in terms of proportion of care hours per patient day deriving from "long" shifts (≥12-hour) for both registered nurses and healthcare assistants.

Results: On 99,043 occasions (53%) observations were significantly delayed, and on 81,568 occasions (44%) observations were missed. Observations were more likely to be delayed when a higher proportion of the hours worked by healthcare assistants were part of long shifts (IRR = 1.05; 95% CI: 1.00-1.10). No significant association was found in relation to the proportion of hours registered nurses worked as long shifts.

Conclusion: On days when a higher proportion of hours worked by health care assistants are from long shifts, the risk of delaying vital signs observations is higher, suggesting lower job performance. While longer shifts are thought to require fewer staff resources to maintain nurse to patient ratios, any benefits may be lost if staff become less productive.

Keywords: Shift Work Schedule; Nursing; 12-hour shifts; Vital Signs Monitoring; Missed Care

For peer review only

For peer review only - http://bmjopen.bmj.com/site/about/guidelines.xhtml

STRENGTHS AND LIMITATIONS OF THIS STUDY

- This study was able to measure an aspect of missed care through an objective outcome, delayed / missed vital signs observations – as opposed to nurse self-reports
- This study drew on a large dataset of nurses' shifts and patients' vital signs observations and covered three years (2012 – 2015)
- However, the study was carried out in a single hospital
- Due to the nature of the administrative datasets available, we were not able to control for staff characteristics including age

For peer review only

For peer review only - http://bmjopen.bmj.com/site/about/guidelines.xhtml

BACKGROUND

Shifts of 12 hours or longer are becoming increasingly common in nursing, based on perceived efficiency savings achieved by reducing the overlap between shorter shifts associated with a three shift system.[1] The same average patients-to-nurse ratio could potentially be kept with a lower number of nursing hours, although claims of improved efficiency are yet to be verified in empirical studies. Furthermore, there is no evidence to suggest that organising the delivery of nursing services in acute hospitals with ≥12-hour (“long”) shifts improves the safety or efficacy of patient care. A recent literature review found no evidence of a beneficial effect of long shifts on patient outcomes,[2] with findings from a number of studies indicating that greater use of long shifts in hospital wards is associated with a number of adverse outcomes, including reducing the quality of care and patient safety;[3 4] higher rates of errors;[5 6] and increased mortality.[7]

Recent studies have examined the impact of long shifts on nurses’ ability to complete all patient care on a given shift. These studies drew on data from 12 European countries and found that nurses working 12-hour shifts were more likely to report higher levels of care being left undone due to lack of time, a phenomenon generally referred to as “missed care”.[3 8] The underlying mechanism for this association has not been studied, although it has been hypothesised that nurses may need to pace during long shifts in order to maintain enough energy until the end of the shift.[9] This would suggest that nurses’ productivity when working 12-hour shifts may be lower, potentially counteracting the expected efficiency savings.

Studies examining the association between long shifts and missed care have relied exclusively on nurse-reported measures, which can result in common-methods bias.[10] Although the direct study of worker performance under different shift systems’ is acknowledged to be challenging,[11] recent expansion in hospital information systems have created new opportunities to study the timely completion of nursing work.[12]

There is now substantial evidence that missed care impacts negatively on patients, with consequences ranging from poor experience of care to increased risk of infection, readmissions and adverse outcomes from undetected physiological deterioration.[13] Timely monitoring and recording of patients’ vital signs is key to identify patient deterioration[14] and electronic recording of these observations provides an opportunity to examine an objective indicator of nurses’ job performance, which has not been previously explored.

For peer review only - http://bmjopen.bmj.com/site/about/guidelines.xhtml

Using routinely collected data, the aim of this study was to investigate the association between the length of shifts worked by nursing staff and adherence to a vital signs observations protocol.

METHODS

This was a retrospective observational study using routinely collected data on nursing staff, shift patterns and patient vital signs observations. Data were extracted for all general adult wards (n= 32) in an acute care hospital Trust in the South of England over a three-year period (1 April 2012- 31 March 2015). These data were obtained as part of a parent study (ISRCTN registration: 17930973 http://www.isrctn.com/ISRCTN17930973). Ethical approval to undertake this research was granted from the University of Southampton Ethics Committee (Submission Number 18311).

Data Sources and variables

Records of worked nursing shifts were extracted from an electronic rostering system implemented hospital-wide and directly linked to payroll. Patient data were extracted from the Patient Administration Systems and were linked to the roster data at the ward level. All data were pseudo-anonymised at source before being provided to the research team.

The nursing workforce is composed of registered nurses (RNs) and unregistered health care assistants (HCAs), also known as healthcare support workers, or “nursing support staff”. For each ward for each day, we calculated registered nurses hours per patient day (RN-HPPD) and healthcare assistants hours per patient day (HCA-HPPD). In each case this was calculated as staff hours / (patient hours/24); a patient day is equivalent to one patient occupying one bed for a full 24 hours. The proportion of care hours per patient day derived from ≥12-hour shifts was calculated for each day in each ward.

Records of vital signs observations were extracted from a database, recorded on hand-held devices at the bedside using the VitalPac™ system. The frequency of vital signs observations required by a patient at any given time is determined by a protocol based on the National Early Warning Score (NEWS), which defines when the next observation is due. Detailed description of NEWS can be found elsewhere;[15] in broad terms, it requires that acutely ill patients are observed more often and defines a minimum observation frequency for a given level of patient acuity.

For peer review only - http://bmjopen.bmj.com/site/about/guidelines.xhtml

For each set of observations, we obtained the expected time to the next observation (based on the protocol) and classified the subsequent observation as delayed (after 1/3 of the defined interval had elapsed) or missed (after 2/3 of the defined interval had elapsed).

According to the hospital escalation protocol, patients are considered at high risk when their NEWS score is six or above, requiring at least four-hourly observations. Since patients with high NEWS scores are those more likely to die and/or to experience critical deterioration[16] we considered only observations of patients who exhibited NEWS ≥ 6. This is the group who are deemed to most require the observations undertaking, and whose outcomes are most vulnerable to deterioration if not sufficiently monitored.

Statistical analysis

The first stage of data analysis involved a descriptive analysis of frequencies and summaries of shift patterns and delayed and missed vital signs observations. The Pearson correlation between the proportion of HPPD deriving from ≥12-hour shifts and missed, and delayed observations was calculated for both registered nurses and healthcare assistants.

Linear mixed-effects Poisson models were then fit. These models sought to examine the effect of higher proportions of registered nurse hours per patient day (RN-HPPD) and healthcare assistant hours per patient day (HCA-HPPD) derived from ≥12-hour shifts on the likelihood of delaying or missing a vital signs observation. Analyses were performed at the ward-day level and delayed/missed vital signs observations were treated as count outcome; the number of observations due was used as an offset. Registered nurse hours per patient day (RN-HPPD) and healthcare assistant hours per patient day (HCA-HPPD) were added as control variables. Intraclass correlation coefficients (ICCs) were computed from unconditional random intercept models to describe the between-ward variation of delayed and missed vital signs observations. The ward level ICC for both delayed and missed observations was 0.37. Due to the high ICC and the data structure (i.e. observations aggregated at the ward-day level), ward was included as a random effect in the multivariate models. As missed care is associated with total staffing levels,[17] we included both RN and HCA-HPPD in all models as control variables. All analysis was undertaken in the R statistical environment version 3.3.2[18] and mixed effects models were fit using the lme4 package.[19]

Patient involvement

The parent project was developed with significant input from local public and patient groups who helped shape the original research focus and with a co-researcher who was actively involved in day-to-day project activities including discussion and interpretation of results.

For peer review only

For peer review only - http://bmjopen.bmj.com/site/about/guidelines.xhtml

RESULTS

In total, there were 390,325 shifts worked by RNs and 268,303 shifts worked by HCAs. The total number of observations in high acuity patients was 184,628. The total number of delayed observations in high acuity patients was 99,043 (53%), while the missed observations were 81,568 (44%), nested in 24,069 ward-days.

Table 1 summarises the distribution of expected, delayed and missed vital signs observations; and of the proportion of HCA and RN-HPPD derived from ≥12-hour shifts.

Table 1 Distribution of missed and delayed observations; and proportion of HPPD deriving from long shifts

	Mean	Median	Interquartile range (IQR)	Min	Max
Expected observations per 24 hours in each ward	7.6	5	2-10	1	87
Delayed observations per 24 hours in each ward	4.1	2	1 – 5	0	56
Missed observations per 24 hours in each ward	3.4	2	1 – 4	0	51
Percentage of HCA-HPPD derived by ≥12-h shifts	46%	57%	0% – 79%	0%	100%
Percentage of RN-HPPD derived by ≥12-h shifts	48%	61%	0% – 82%	0%	100%

The mean number of delayed observations in each ward-day was 4.1, while on average 3.4 observations were missed on each ward-day. Across the three years, on average 46% of HCA-HPPD derived from ≥12-hour shifts, with large variation (Interquartile range (IQR) 0% – 79%) and on average 48% of RN-HPPD derived from ≥12-hour shifts (IQR 0% –82%).

The proportion of RN-HPPD contributed by ≥12-hour shifts was positively correlated with the rate of both delayed ($r = 0.26$) and missed observations ($r = 0.25$). Similarly, the proportion

For peer review only - http://bmjopen.bmj.com/site/about/guidelines.xhtml

of HCA-HPPD deriving from ≥12-hour shifts was also correlated with delayed (r = 0.27) and missed observations (r = 0.27).

Table 2 Multivariable association between the proportions of RN-HPPD, HCA-HPPD deriving from 12 hours or more shifts and delayed/missed vital signs observations

**Delayed vital signs observations**		
**Shift characteristics**	**IRR†**	**95% CI**
Proportion of RN-HPPD deriving from ≥12-h shifts	0.96	0.91-1.01
Proportion of HCA-HPPD deriving from ≥12-h shifts	1.05*	1.00-1.10
**Missed vital signs observations**		
**Shift characteristics**	**IRR**	**95% CI**
Proportion of RN-HPPD deriving from ≥12-h shifts	0.96	0.91-1.01
Proportion of HCA-HPPD deriving from ≥12-h shifts	1.04	0.99-1.09

* Statistically significant at $p < 0.05$

† Incidence Rate Ratio (all models include random effect for ward and RN / HCA HPPD)

Random effect: ward

In multivariate models controlling for staffing levels, the proportion of HCA-HPPD derived by ≥12-hour shifts was significantly associated with rate of delayed vital signs observations. An additional 1% of HCA-HPPD derived from long shifts was associated with a 5% increase in the rate of delayed observations compared to days where no long shifts were worked by HCAs (IRR = 1.05; 95% CI: 1.00-1.10). Results for missed observations were similar but not statistically significant. When looking at registered nurses, the proportion of hours worked through long shifts by RNs was not significantly associated with neither delayed nor missed vital signs observations.

DISCUSSION

This is the first study to explore the relationship between long nursing shifts and an objective measure of missed care in terms of missed/delayed vital signs observations. We found that delays in obtaining vital signs observations were relatively common for patients assessed as

For peer review only - http://bmjopen.bmj.com/site/about/guidelines.xhtml

requiring frequent observation. For HCAs, where more of their hours were worked as long shifts, there was a significant increase in delayed vital signs observations. We did not find a significant relationship between delayed/missed vital signs observations and the proportion of RN HPPD worked as part of long shifts.

In the context of a growing body of research suggesting that long nursing shifts on hospital wards are associated with lower quality of care and worse patient outcomes, this finding points to one possible mechanism, as using vital signs observations as an indicator, job performance would appear to be lower for HCAs working longer shifts. This in turn is likely to be a product of the increased fatigue that is frequently reported by those working long shifts.[20 21]

Previous research suggesting that the job performance of nursing staff may reduce when working 12-hour shifts has focussed specifically on RNs,[3 9 20] but we found no significant association for this staff group. These studies have relied on general reports of missed care whereas we focussed specifically on vital signs observations. Evidence of the association between staffing levels and missed care shows that, in general, RNs are more likely to report that lack of time causes them to miss interpersonal care (e.g. talking and comforting patients and planning patient care) than “clinical” care.[17] This may reflect a prioritisation of some aspects of care by RNs in the face of limited capacity. It might be that other aspects of RN work are adversely affected by 12-hour shifts, but observation of patient groups identified as at risk of deterioration remains a priority.

The monitoring of vital signs is increasingly being delegated to HCAs. Research undertaken with registered nurses and healthcare assistants in the UK found that vital signs observations were a central feature of healthcare assistants’ roles.[22] Furthermore, there are reports of registered nurses considering the assessment of vital signs a basic task and therefore one that RNs may delegate to less qualified staff members.[23] The extent to which such delegation has occurred in the present Trust is unclear, as the record of who undertook the observation was incomplete and unreliable, although all health care assistants are expected to have the competency to undertake and record vital signs observations. Anecdotal accounts suggest that HCAs undertaking vital signs is widespread. Thus, it may simply be that compliance with the vital signs observation protocol is a good indicator of job performance for HCAs but not for RNs because of different responsibilities. Alternatively, it may be that long shifts adversely affect HCAs but not RNs or that RNs place a higher priority on ensuring timely observation even though their overall capacity is similarly affected by long shifts.

For peer review only - http://bmjopen.bmj.com/site/about/guidelines.xhtml

For peer review only

This study has some limitations. We were only able to focus on a single aspect of job performance - vital signs monitoring - which may not be reflective of global performance. We were also unable to ascertain which staff group was assigned to take observations. Furthermore, it was not possible to determine whether observations were deliberately omitted for clinical reasons despite being in contravention of the protocol. For example, in a previous study nursing staff have reported choosing not to perform some observations at night because they felt waking patients could have a negative effect on individual patients. These reasons included patients who had difficulty sleeping and 'confused' patients who may become agitated.[24] We did not study the direct consequences of missed vital signs observations, although other studies have shown that missed care in general is associated with adverse patient outcomes.[13] Recent studies have shown that nurse-reported missed care mediates the relationship between low staffing and mortality,[25] with a further study showing specifically that missed vital signs observations mediated an association between RN staffing levels and mortality.[26]

We were not able to account for staff characteristics including age and personal commitments external to work, both of which may influence job performance. This study was conducted in a single site, and these findings may not generalise to other NHS Trusts. However, a previous study found that shift patterns tend to vary more within hospitals than between hospitals in England.[3]

CONCLUSIONS

This is the first study in nursing to use objective measures of shift patterns and missed care obtained from records spanning a long period of time. Where working hours involved a larger proportion of long shifts delays in obtaining scheduled vital signs observations by nursing support staff were observed. While this study lends partial support to previous findings using global self-report measures, further research should explore better measures of job performance and more complete data on costs, and effects, to work out which shift patterns are most cost-effective for ward-based services.

For peer review only - http://bmjopen.bmj.com/site/about/guidelines.xhtml

ACKNOWLEDGMENTS

The authors thank Portsmouth Hospitals NHS Trust for allowing us to use the study data.

FUNDING

This work was supported by NIHR Collaboration for Leadership in Applied Health Research and Care Wessex and by the NIHR HS&DR – grant number - 13/114/17: “Nurse staffing levels, missed vital signs observations and mortality in hospital wards: modelling the consequences and costs of variations in nurse staffing and skill mix”. The views expressed in this publication are those of the authors and not necessarily those of the NHS, the National Institute for Health Research or the Department of Health and Social Care.

CONFLICT OF INTEREST

The authors have no conflict of interest to declare.

AUTHORS CONTRIBUTIONS

CDO drafted the article, giving substantial contributions to its conception and design, collaborated in acquiring the data, performed statistical analyses and interpreted the data, and gave final approval of the version to be published. She agreed to be accountable for all aspects of the work in ensuring that questions related to the accuracy or integrity of any part of the work are appropriately investigated and resolved. PG gave substantial contributions to the conception and design of the work, the interpretation of data and revised critically the article for important intellectual content, and gave final approval of the version to be published. OR gave substantial contributions to acquiring the data, to the statistical analysis and data interpretation, and revised critically the article for important intellectual content, and gave final approval of the version to be published. AR-S collaborated in the conception of the study, revised critically the article for important intellectual content and gave final approval of the version to be published. PM gave substantial contributions to acquiring the data, collaborated with statistical analysis and data interpretation, revised critically the article for important intellectual content and gave final approval of the version to be published. JB gave substantial contributions to the conception of the study, revised critically the article for important intellectual content and gave final approval of the version to be published.

For peer review only

DATA SHARING AGREEMENT

Due to the nature of this study and the conditions attached to original data agreements, there is no data available for wider use. All queries should be submitted to the corresponding author in the first instance.

For peer review only

For peer review only - http://bmjopen.bmj.com/site/about/guidelines.xhtml

REFERENCES

1. NHS Evidence. Moving to 12-hour shift patterns: to increase continuity and reduce costs, 2010.

2. Dall'Ora C, Ball J, Recio-Saucedo A, et al. Characteristics of shift work and their impact on employee performance and wellbeing: A literature review. *Int J Nurs Stud* 2016;57:12-27. doi: 10.1016/j.ijnurstu.2016.01.007

3. Griffiths P, Dall'Ora C, Simon M, et al. Nurses' shift length and overtime working in 12 European countries: the association with perceived quality of care and patient safety. *Med Care* 2014;52(11):975-81. doi: 10.1097/MLR.0000000000000233

4. Stimpfel AW, Aiken LH. Hospital staff nurses' shift length associated with safety and quality of care. *J Nurs Care Qual* 2013;28(2):122-9. doi: 10.1097/NCQ.0b013e3182725f09

5. Rogers AE, Hwang WT, Scott LD, et al. The working hours of hospital staff nurses and patient safety. *Health Aff (Millwood)* 2004;23(4):202-12. doi: 10.1377/hlthaff.23.4.202 [published Online First: 2004/08/21]

6. Clendon J, Gibbons V. 12 h shifts and rates of error among nurses: A systematic review. *Int J Nurs Stud* 2015;52(7):1231-42. doi: 10.1016/j.ijnurstu.2015.03.011 [published Online First: 2015/04/26]

7. Trinkoff AM, Johantgen M, Storr CL, et al. Nurses' work schedule characteristics, nurse staffing, and patient mortality. *Nurs Res* 2011;60(1):1-8. doi: 10.1097/NNR.0b013e3181fff15d [published Online First: 2010/12/04]

8. Ball J, Day T, Murrells T, et al. Cross-sectional examination of the association between shift length and hospital nurses job satisfaction and nurse reported quality measures. *BMC Nurs* 2017;16(1):26. doi: 10.1186/s12912-017-0221-7

9. Reid N, Robinson G, Todd C. The quantity of nursing care on wards working 8- and 12-hour shifts. *Int J Nurs Stud* 1993;30(5):403-13. doi: https://doi.org/10.1016/0020-7489(93)90050-5

10. Antonakis J, Bendahan S, Jacquart P, et al. On making causal claims: A review and recommendations. *Leadership Quart* 2010;21(6):1086-120. doi: 10.1016/j.leaqua.2010.10.010

For peer review only - http://bmjopen.bmj.com/site/about/guidelines.xhtml

11. Folkard S, Tucker P. Shift work, safety and productivity. *Occup Med (Lond)* 2003;53(2):95-101. doi: 10.1093/occmed/kqg047

12. Griffiths P, Ball J, Drennan J, et al. Nurse staffing and patient outcomes: Strengths and limitations of the evidence to inform policy and practice. A review and discussion paper based on evidence reviewed for the National Institute for Health and Care Excellence Safe Staffing guideline development. *Int J Nurs Stud* 2016;63:213-25. doi: 10.1016/j.ijnurstu.2016.03.012

13. Recio-Saucedo A, Dall'Ora C, Maruotti A, et al. What impact does nursing care left undone have on patient outcomes? Review of the literature. *J Clin Nurs* 2017;0(0) doi: doi:10.1111/jocn.14058

14. Smith GB. In-hospital cardiac arrest: is it time for an in-hospital 'chain of prevention'? *Resuscitation* 2010;81(9):1209-11. doi: 10.1016/j.resuscitation.2010.04.017 [published Online First: 2010/07/06]

15. Royal College of Physicians. National Early Warning (NEWS). Standardising the assessment of acute-illness severity in the NHS. London: Royal College of Physicians, 2012.

16. Smith GB, Prytherch DR, Meredith P, et al. The ability of the National Early Warning Score (NEWS) to discriminate patients at risk of early cardiac arrest, unanticipated intensive care unit admission, and death. *Resuscitation* 2013;84(4):465-70. doi: 10.1016/j.resuscitation.2012.12.016

17. Griffiths P, Recio-Saucedo A, Dall'Ora C, et al. The association between nurse staffing and omissions in nursing care: A systematic review. *J Adv Nurs* 2018;0(0) doi: doi:10.1111/jan.13564

18. R: A language and environment for statistical computing [program]. 1.0.136 version. Vienna, Austria: R Foundation for Statistical Computing, 2017.

19. lme4: Linear Mixed-Effects Models Using Eigen and S4 [program]. 1.1-13 version, 2015.

20. Barker LM, Nussbaum MA. Fatigue, performance and the work environment: a survey of registered nurses. *J Adv Nurs* 2011;67(6):1370-82. doi: 10.1111/j.1365-2648.2010.05597.x

21. Chen J, Davis KG, Daraiseh NM, et al. Fatigue and recovery in 12-hour dayshift hospital nurses. *J Nurs Manag* 2014;22(5):593-603. doi: 10.1111/jonm.12062

For peer review only - http://bmjopen.bmj.com/site/about/guidelines.xhtml

22. Bach S, Kessler I, Heron P. Nursing a Grievance? The Role of Healthcare Assistants in a Modernized National Health Service. *Gender, Work & Organization* 2012;19(2):205-24. doi: 10.1111/j.1468-0432.2009.00502.x

23. Hogan J. Why don't nurses monitor the respiratory rates of patients? *Br J Nurs* 2006;15(9):489-92. doi: 10.12968/bjon.2006.15.9.21087

24. Hope J, Recio-Saucedo A, Fogg C, et al. A fundamental conflict of care: Nurses' accounts of balancing patients' sleep with taking vital sign observations at night. *J Clin Nurs* 2017;0(0) doi: 10.1111/jocn.14234

25. Ball JE, Bruyneel L, Aiken LH, et al. Post-operative mortality, missed care and nurse staffing in nine countries: A cross-sectional study. *Int J Nurs Stud* 2018;78:10-15. doi: 10.1016/j.ijnurstu.2017.08.004

26. Griffiths P, Ball J, Bloor K, et al. Nurse staffing levels, missed vital signs observations and mortality in hospital wards: retrospective longitudinal observational study using routinely collected data - Final report. *Health Services and Delivery Research* 2018

For peer review only

For peer review only - http://bmjopen.bmj.com/site/about/guidelines.xhtml

STROBE 2007 (v4) Statement—Checklist of items that should be included in reports of *cross-sectional studies*

Section/Topic	Item #	Recommendation	Reported on page #
**Title and abstract**	1	(*a*) Indicate the study's design with a commonly used term in the title or the abstract	1
		(*b*) Provide in the abstract an informative and balanced summary of what was done and what was found	3
**Introduction**			
Background/rationale	2	Explain the scientific background and rationale for the investigation being reported	5
Objectives	3	State specific objectives, including any prespecified hypotheses	6
**Methods**			
Study design	4	Present key elements of study design early in the paper	6
Setting	5	Describe the setting, locations, and relevant dates, including periods of recruitment, exposure, follow-up, and data collection	6-7
Participants	6	(*a*) Give the eligibility criteria, and the sources and methods of selection of participants	6-7
Variables	7	Clearly define all outcomes, exposures, predictors, potential confounders, and effect modifiers. Give diagnostic criteria, if applicable	6-7
Data sources/ measurement	8*	For each variable of interest, give sources of data and details of methods of assessment (measurement). Describe comparability of assessment methods if there is more than one group	6-7
Bias	9	Describe any efforts to address potential sources of bias	6-7
Study size	10	Explain how the study size was arrived at	6-7
Quantitative variables	11	Explain how quantitative variables were handled in the analyses. If applicable, describe which groupings were chosen and why	6-7
Statistical methods	12	(*a*) Describe all statistical methods, including those used to control for confounding	7
		(*b*) Describe any methods used to examine subgroups and interactions	N/A
		© Explain how missing data were addressed	N/A
		(*d*) If applicable, describe analytical methods taking account of sampling strategy	N/A
		€ Describe any sensitivity analyses	N/A
**Results**			

For peer review only

Participants	13*	(a) Report numbers of individuals at each stage of study—eg numbers potentially eligible, examined for eligibility, confirmed eligible, included in the study, completing follow-up, and analysed	8
		(b) Give reasons for non-participation at each stage	N/A
		© Consider use of a flow diagram	N/A
Descriptive data	14*	(a) Give characteristics of study participants (eg demographic, clinical, social) and information on exposures and potential confounders	8
		(b) Indicate number of participants with missing data for each variable of interest	N/A
Outcome data	15*	Report numbers of outcome events or summary measures	8
Main results	16	(*a*) Give unadjusted estimates and, if applicable, confounder-adjusted estimates and their precision (eg, 95% confidence interval). Make clear which confounders were adjusted for and why they were included	9
		(*b*) Report category boundaries when continuous variables were categorized	N/A
		© If relevant, consider translating estimates of relative risk into absolute risk for a meaningful time period	N/A
Other analyses	17	Report other analyses done—eg analyses of subgroups and interactions, and sensitivity analyses	N/A
**Discussion**			
Key results	18	Summarise key results with reference to study objectives	9
Limitations	19	Discuss limitations of the study, taking into account sources of potential bias or imprecision. Discuss both direction and magnitude of any potential bias	10-11
Interpretation	20	Give a cautious overall interpretation of results considering objectives, limitations, multiplicity of analyses, results from similar studies, and other relevant evidence	10-11
Generalisability	21	Discuss the generalisability (external validity) of the study results	11
**Other information**			
Funding	22	Give the source of funding and the role of the funders for the present study and, if applicable, for the original study on which the present article is based	3

*Give information separately for cases and controls in case-control studies and, if applicable, for exposed and unexposed groups in cohort and cross-sectional studies.

Note: An Explanation and Elaboration article discusses each checklist item and gives methodological background and published examples of transparent reporting. The STROBE checklist is best used in conjunction with this article (freely available on the Web sites of PLoS Medicine at http://www.plosmedicine.org/, Annals of Internal Medicine at http://www.annals.org/, and Epidemiology at http://www.epidem.com/). Information on the STROBE Initiative is available at www.strobe-statement.org.

For peer review only

BMJ Open

Nurses' 12-hour shifts and missed or delayed vital signs observations on hospital wards: retrospective observational study

Journal:	*BMJ Open*
Manuscript ID	bmjopen-2018-024778.R1
Article Type:	Research
Date Submitted by the Author:	10-Oct-2018
Complete List of Authors:	Dall'Ora, Chiara; University of Southampton, Faculty of Health Sciences; NIHR Collaboration for Leadership in Applied Health Research and Care Wessex,<br>Griffiths, Peter; University of Southampton, Faculty of Health Sciences<br>Redfern, Oliver; University of Portsmouth , School of Computing; University of Oxford, Nuffield Department of Clinical Neurosciences<br>Recio-Saucedo, Alejandra; University of Southampton, Faculty of Health Sciences<br>Meredith, Paul; Portsmouth Hospitals NHS Trust, Research & Innovation Department<br>Ball, Jane; National Institute for Health Research Collaboration for Leadership in Applied Health Research and Care (Wessex),University of Southampton, Centre for Innovation and Leadership in Health Sciences
**Primary Subject Heading**:	Health services research
Secondary Subject Heading:	Nursing
Keywords:	Shift Work Schedule, Nursing, 12-hour Shifts, Vital Signs Monitoring, Missed Care

SCHOLARONE™
Manuscripts

TITLE PAGE

Nurses' 12-hour shifts and missed or delayed vital signs observations on hospital wards: retrospective observational study

Chiara Dall'Ora* [1,2] RN, MSc, PhD, University of Southampton, Building 67, Highfield, Southampton, SO17 1BJ, UK, C.Dall'ora@soton.ac.uk, +44(023) 8059 8249

Peter Griffiths*[1,2], Oliver Redfern[3,4], Alejandra Recio-Saucedo[2], Paul Meredith[5], Jane Ball[2], On behalf of the Missed Care Study Group†

* Chiara Dall'Ora and Peter Griffiths contributed equally to this manuscript

[1] National Institute for Health Research Collaboration for Leadership in Applied Health Research and Care (Wessex), University of Southampton, Southampton, UK

[2] Faculty of Health Sciences, University of Southampton, Southampton, UK

[3] School of Computing, University of Portsmouth, UK

[4] Kadoorie Centre for Critical Care Research and Education, Nuffield Department of Clinical Neurosciences, University of Oxford, UK

[5] Research & Innovation Department, Portsmouth Hospitals NHS Trust, Portsmouth, UK

† The Missed Care Study Group comprises Karen Bloor, University of York; Dankmar Böhning, University of Southampton; Jim Briggs, University of Portsmouth, Centre for Healthcare Modelling and Informatics; Anya De Iongh, Independent lay researcher; Jeremy Jones, University of Southampton, Faculty of Health Sciences; Caroline Kovacs, University of Portsmouth, Centre for Healthcare Modelling and Informatics; David Prytherch University of Portsmouth, Centre for Healthcare Modelling and Informatics and Portsmouth Hospitals NHS Trust, Clinical Outcomes Research Group; Paul Schmidt, National Institute for Health Research

For peer review only - http://bmjopen.bmj.com/site/about/guidelines.xhtml

Collaboration for Applied Health Research and Care (Wessex) and Portsmouth Hospitals NHS Trust, Clinical Outcomes Research Group; Nicky Sinden, Portsmouth Hospitals NHS Trust, Clinical Outcomes Research Group; Gary Smith, Bournemouth University

Word count: 2741

For peer review only

For peer review only - http://bmjopen.bmj.com/site/about/guidelines.xhtml

ABSTRACT

Objectives: 12-hour shifts worked by nurses on acute hospital wards have been associated with increased rates of missed care reported by nurses. This study aimed to measure the association between nurses working shifts of at least 12 hours and an objective measure of missed care – vital signs observations taken on time according to an acuity-based surveillance protocol.

Design: A retrospective observational study using routinely collected data from March 2012 to March 2015.

Setting: 32 general inpatient wards at a large acute hospital in England.

Participants: 658,628 nursing shifts nested in 24,069 ward-days.

Outcome measures: The rate of daily delayed and missed vital signs observations. We focussed on situations where vital signs observations were required at least every 4 hours and measured the number of instances where observations were delayed or missed, per 24-hour period. For each ward and each day, shift patterns were characterised in terms of proportion of care hours per patient day deriving from "long" shifts (≥12-hour) for both registered nurses and healthcare assistants.

Results: On 99,043 occasions (53%) observations were significantly delayed, and on 81,568 occasions (44%) observations were missed. Observations were more likely to be delayed when a higher proportion of the hours worked by healthcare assistants were part of long shifts (IRR = 1.05; 95% CI: 1.00-1.10). No significant association was found in relation to the proportion of hours registered nurses worked as long shifts.

Conclusion: On days when a higher proportion of hours worked by health care assistants are from long shifts, the risk of delaying vital signs observations is higher, suggesting lower job performance. While longer shifts are thought to require fewer staff resources to maintain nurse to patient ratios, any benefits may be lost if staff become less productive.

Keywords: Shift Work Schedule; Nursing; 12-hour shifts; Vital Signs Monitoring; Missed Care

For peer review only

STRENGTHS AND LIMITATIONS OF THIS STUDY

- This study was able to measure an aspect of missed care through an objective outcome, delayed / missed vital signs observations – as opposed to nurse self-reports
- This study drew on a large dataset of nurses' shifts and patients' vital signs observations and covered three years (2012 – 2015)
- However, the study was carried out in a single hospital
- Due to the nature of the administrative datasets available, we were not able to control for staff characteristics including age

For peer review only

For peer review only - http://bmjopen.bmj.com/site/about/guidelines.xhtml

BACKGROUND

Shifts of 12 hours or longer are becoming increasingly common in nursing, based on perceived efficiency savings achieved by reducing the overlap between shorter shifts associated with a three shift system.[1] The same average patients-to-nurse ratio could potentially be kept with a lower number of nursing hours, although claims of improved efficiency are yet to be verified in empirical studies. Furthermore, there is no evidence to suggest that organising the delivery of nursing services in acute hospitals with ≥12-hour ("long") shifts improves the safety or efficacy of patient care. A recent literature review found no evidence of a beneficial effect of long shifts on patient outcomes,[2] with findings from a number of studies indicating that greater use of long shifts in hospital wards is associated with a number of adverse outcomes, including reducing the quality of care and patient safety;[3 4] higher rates of errors;[5 6] and increased mortality.[7]

Recent studies have examined the impact of long shifts on nurses' ability to complete all patient care on a given shift. These studies drew on data from 12 European countries and found that nurses working 12-hour shifts were more likely to report higher levels of care being left undone due to lack of time, a phenomenon generally referred to as "missed care".[8] The underlying mechanism for this association has not been studied, although it has been hypothesised that nurses may need to pace during long shifts in order to maintain enough energy until the end of the shift.[9] This would suggest that nurses' productivity when working 12-hour shifts may be lower, potentially counteracting the expected efficiency savings.

Studies examining the association between long shifts and missed care have relied exclusively on nurse-reported measures, which can result in common-methods bias.[10] Although the direct study of worker performance under different shift systems' is acknowledged to be challenging,[11] recent expansion in hospital information systems have created new opportunities to study the timely completion of nursing work.[12]

There is now substantial evidence that missed care impacts negatively on patients, with consequences ranging from poor experience of care to increased risk of infection, readmissions and adverse outcomes from undetected physiological deterioration.[13] Timely monitoring and recording of patients' vital signs is key to identify patient deterioration[14] and electronic recording of these observations provides an opportunity to examine an objective indicator of nurses' job performance, which has not been previously explored.

For peer review only - http://bmjopen.bmj.com/site/about/guidelines.xhtml

Using routinely collected data, the aim of this study was to investigate the association between the length of shifts worked by nursing staff and adherence to a vital signs observations protocol.

METHODS

This was a retrospective observational study using routinely collected data on nursing staff, shift patterns and patient vital signs observations. Data were extracted for all general adult wards (n= 32) in an acute care hospital Trust in the South of England over a three-year period (1 April 2012- 31 March 2015). These data were obtained as part of a parent study (ISRCTN registration: 17930973 http://www.isrctn.com/ISRCTN17930973). Ethical approval to undertake this research was granted from the University of Southampton Ethics Committee (Submission Number 18311).

Data Sources and variables

Records of worked nursing shifts were extracted from an electronic rostering system implemented hospital-wide and directly linked to payroll. Patient data were extracted from the Patient Administration Systems and were linked to the roster data at the ward level. The patient sample included all patients who were on the wards in the days included in the analysis. All data were pseudo-anonymised at source before being provided to the research team.

The nursing workforce is composed of registered nurses (RNs) and unregistered health care assistants (HCAs), also known as healthcare support workers, or "nursing support staff". For each ward for each day, we calculated registered nurses hours per patient day (RN-HPPD) and healthcare assistants hours per patient day (HCA-HPPD). In each case this was calculated as staff hours / (patient hours/24); a patient day is equivalent to one patient occupying one bed for a full 24 hours. The proportion of care hours per patient day derived from ≥12-hour shifts was calculated for each day in each ward.

Vital signs observations were derived from a database of records made using the VitalPac™ system, where nurses record clinical data on hand held devices at the bedside. These systems comprise the only official record of observations that are maintained on the wards in the study and the use of the system is mandated by Trust policy. The frequency of vital signs observations required by a patient at any given time is determined by a protocol based on the National Early Warning Score (NEWS), which defines when the next observation is due.

For peer review only - http://bmjopen.bmj.com/site/about/guidelines.xhtml

Detailed description of NEWS can be found elsewhere;[15] in broad terms, it requires that acutely ill patients are observed more often and defines a minimum observation frequency for a given level of patient acuity.

For each set of observations, we obtained the expected time to the next observation (based on the protocol) and classified the subsequent observation as delayed (after 1/3 of the defined interval had elapsed) or missed (after 2/3 of the defined interval had elapsed). This classification was arrived at after extensive discussion, examination of the distribution of delays in taking observations and consultation with patient and professional stakeholders. The classification of missed observations reflects that an observation taken late, but near to the time a subsequent observation is due, is more clearly fulfilling the second scheduled observation than the first.

According to the hospital escalation protocol, patients are considered at high risk when their NEWS score is six or above, requiring observations at least once every four hours. Since patients with high NEWS scores are those more likely to die and/or to experience critical deterioration[16] we considered only observations of patients who exhibited NEWS ≥ 6. This is the group who are deemed to most require the observations undertaking, and whose outcomes are most vulnerable to deterioration if not sufficiently monitored.

Statistical analysis

The first stage of data analysis involved a descriptive analysis of frequencies and summaries of shift patterns and delayed and missed vital signs observations. The Pearson correlation between the proportion of HPPD deriving from ≥12-hour shifts and missed, and delayed observations was calculated for both registered nurses and healthcare assistants.

Linear mixed-effects Poisson models were then fit. These models sought to examine the effect of higher proportions of registered nurse hours per patient day (RN-HPPD) and healthcare assistant hours per patient day (HCA-HPPD) derived from ≥12-hour shifts on the likelihood of delaying or missing a vital signs observation. Analyses were performed at the ward-day level and delayed/missed vital signs observations were treated as count outcome; the number of observations due was used as an offset. Registered nurse hours per patient day (RN-HPPD) and healthcare assistant hours per patient day (HCA-HPPD) were added as control variables. Intraclass correlation coefficients (ICCs) were computed from unconditional random intercept models to describe the between-ward variation of delayed and missed vital signs observations. The ward level ICC for both delayed and missed observations was 0.37. Due to the high ICC and the data structure (i.e. observations aggregated at the ward-day level), ward was included

For peer review only - http://bmjopen.bmj.com/site/about/guidelines.xhtml

as a random effect in the multivariate models. As missed care is associated with total staffing levels,[17] we included both RN and HCA-HPPD in all models as control variables. All analysis was undertaken in the R statistical environment version 3.3.2[18] and mixed effects models were fit using the lme4 package.[19]

Patient involvement

The parent project was developed with significant input from local public and patient groups who helped shape the original research focus and with a co-researcher who was actively involved in day-to-day project activities including discussion and interpretation of results. As part of the parent study, we undertook a series of consultations with public, patient and clinical experts in order to identify issues that should be considered, including determining an acceptable level of vital signs observations compliance (completing a full set of observations in time). The work was completed in stages so that different groups were approached separately to share views relevant to each individual group. Our approach, co-designed with the parent's study PPI representative / lay co-researcher was intended to make the key issues to be discussed tangible for a more lay audience and professional stakeholders such as staff nurses.

RESULTS

There were 1944 nursing staff members in the sample. 1244 staff members were registered nurses and 700 staff members were classified as healthcare assistants. Nursing staff worked on average 276 shifts within the three-year study period. In total, 390,325 shifts were worked by RNs and 268,303 shifts were worked by HCAs. The patient population comprised of 142,046 patients; their mean age was 67; the majority (79%) were emergency admissions, and 50% of the patients had no comorbidities (Charlson comorbidity index = 0). The total number of observations in high acuity patients was 184,628. The total number of delayed observations in high acuity patients was 99,043 (53%), while the missed observations were 81,568 (44%), nested in 24,069 ward-days. Wards differed as regards size (i.e. number of beds), mean HPPD and mean skill mix (i.e. the proportion of RN-HPPD over the total HPPD, calculated as RN-HPPD/(RN-HPPD + HCA-HPPD)). For a detailed description of ward characteristics, please see Appendix 1.

Table 1 summarises the distribution of expected, delayed and missed vital signs observations; and of the proportion of HCA and RN-HPPD derived from ≥12-hour shifts.

For peer review only - http://bmjopen.bmj.com/site/about/guidelines.xhtml

Table 1 Distribution of missed and delayed observations; and proportion of HPPD deriving from long shifts

	Mean	Median	Interquartile range (IQR)	Min	Max
Expected observations per 24 hours in each ward	7.6	5	2-10	1	87
Delayed observations per 24 hours in each ward	4.1	2	1 – 5	0	56
Missed observations per 24 hours in each ward	3.4	2	1 – 4	0	51
Percentage of HCA-HPPD derived by ≥12-h shifts	46%	57%	0% – 79%	0%	100%
Percentage of RN-HPPD derived by ≥12-h shifts	48%	61%	0% – 82%	0%	100%

The mean number of delayed observations in each ward-day was 4.1, while on average 3.4 observations were missed on each ward-day. Across the three years, on average 46% of HCA-HPPD derived from ≥12-hour shifts, with large variation (Interquartile range (IQR) 0% –79%) and on average 48% of RN-HPPD derived from ≥12-hour shifts (IQR 0% –82%).

The proportion of RN-HPPD contributed by ≥12-hour shifts was positively correlated with the rate of both delayed (r = 0.26) and missed observations (r = 0.25). Similarly, the proportion of HCA-HPPD deriving from ≥12-hour shifts was also correlated with delayed (r = 0.27) and missed observations (r = 0.27). Table 2 reports the association between the proportion of RN-HCPPD and HCA-HCPPD deriving from ≥ 12-hour shifts and delayed and missed vital signs observations.

For peer review only - http://bmjopen.bmj.com/site/about/guidelines.xhtml

Table 2 Multivariable association between the proportions of RN-HPPD, HCA-HPPD deriving from 12 hours or more shifts and delayed/missed vital signs observations

**Delayed vital signs observations**		
**Shift characteristics**	**IRR†**	**95% CI**
Proportion of RN-HPPD deriving from ≥12-h shifts	0.96	0.91-1.01
Proportion of HCA-HPPD deriving from ≥12-h shifts	1.05*	1.00-1.10
**Missed vital signs observations**		
**Shift characteristics**	**IRR**	**95% CI**
Proportion of RN-HPPD deriving from ≥12-h shifts	0.96	0.91-1.01
Proportion of HCA-HPPD deriving from ≥12-h shifts	1.04	0.99-1.09

* Statistically significant at $p< 0.05$
† Incidence Rate Ratio (all models include random effect for ward and RN / HCA HPPD)
Random effect: ward

In multivariate models controlling for staffing levels, the proportion of HCA-HPPD derived by ≥12-hour shifts was significantly associated with rate of delayed vital signs observations. An additional 1% of HCA-HPPD derived from long shifts was associated with a 5% increase in the rate of delayed observations compared to days where no long shifts were worked by HCAs (IRR = 1.05; 95% CI: 1.00-1.10). Results for missed observations were similar but not statistically significant. When looking at registered nurses, the proportion of hours worked through long shifts by RNs was not significantly associated with neither delayed nor missed vital signs observations.

To exclude the possibility that the use of ≥12-hour shifts was associated with increased workload of more acute patients, we ran a linear mixed model to establish how proportions of acutely ill patients (NEWS ≥6) on a ward-day were associated with proportions of RN-HPPD and HCA-HPPD deriving from 12-hour shifts, and we found no association (results available from authors).

For peer review only - http://bmjopen.bmj.com/site/about/guidelines.xhtml

DISCUSSION

This is the first study to explore the relationship between long nursing shifts and an objective measure of missed care in terms of missed/delayed vital signs observations. We found that delays in obtaining vital signs observations were relatively common for patients assessed as requiring frequent observation. For HCAs, where more of their hours were worked as long shifts, there was a significant increase in delayed vital signs observations. We did not find a significant relationship between delayed/missed vital signs observations and the proportion of RN HPPD worked as part of long shifts.

In the context of a growing body of research suggesting that long nursing shifts on hospital wards are associated with lower quality of care and worse patient outcomes, this finding points to one possible mechanism, as using vital signs observations as an indicator, job performance would appear to be lower for HCAs working longer shifts. This in turn is likely to be a product of the increased fatigue that is frequently reported by those working long shifts.[20 21]

Previous research suggesting that the job performance of nursing staff may reduce when working 12-hour shifts has focussed specifically on RNs,[4 9 20] but we found no significant association for this staff group. These studies have relied on general reports of missed care whereas we focussed specifically on vital signs observations. Evidence of the association between staffing levels and missed care shows that, in general, RNs are more likely to report that lack of time causes them to miss interpersonal care (e.g. talking and comforting patients and planning patient care) than "clinical" care.[17] This may reflect a prioritisation of some aspects of care by RNs in the face of limited capacity. It might be that other aspects of RN work are adversely affected by 12-hour shifts, but observation of patient groups identified as at risk of deterioration remains a priority.

The monitoring of vital signs is increasingly being delegated to HCAs. Research undertaken with registered nurses and healthcare assistants in the UK found that vital signs observations were a central feature of healthcare assistants' roles.[22] Furthermore, there are reports of registered nurses considering the assessment of vital signs a basic task and therefore one that RNs may delegate to less qualified staff members.[23] The extent to which such delegation has occurred in the present Trust is unclear, as the record of who undertook the observation was incomplete and unreliable, although all health care assistants are expected to have the competency to undertake and record vital signs observations. Being unable to establish whether

For peer review only - http://bmjopen.bmj.com/site/about/guidelines.xhtml

such delegation occurs prevents us from understanding the role HCAs may play in avoiding nursing missed care. Anecdotal accounts suggest that HCAs undertaking vital signs is widespread. Thus, it may simply be that compliance with the vital signs observation protocol is a good indicator of job performance for HCAs but not for RNs because of different responsibilities. Alternatively, it may be that long shifts adversely affect HCAs but not RNs or that RNs place a higher priority on ensuring timely observation even though their overall capacity is similarly affected by long shifts.

This study has some limitations. We were only able to focus on a single aspect of job performance - vital signs monitoring - which may not be reflective of global performance. We were also unable to ascertain which staff group was assigned to take observations. Furthermore, it was not possible to determine whether observations were deliberately omitted for clinical reasons despite being in contravention of the protocol. For example, in a previous study nursing staff have reported choosing not to perform some observations at night because they felt waking patients could have a negative effect on individual patients. These reasons included patients who had difficulty sleeping and 'confused' patients who may become agitated.[24] Furthermore, patients may not have been available at the time observations were due, for example if they were undergoing diagnostic tests (e.g. radiology) elsewhere in the hospital. We did not study the direct consequences of missed vital signs observations, although other studies have shown that missed care in general is associated with adverse patient outcomes.[13] Recent studies have shown that nurse-reported missed care mediates the relationship between low staffing and mortality,[25] with a further study showing specifically that missed vital signs observations mediated an association between RN staffing levels and mortality.[26]

We were not able to account for staff characteristics including age and personal commitments external to work, both of which may influence job performance. Furthermore, in common with several previous missed care studies,[17] we weren't able to obtain a nurse-reported reason for missing vital signs observations. This study was conducted in a single site, and these findings may not generalise to other NHS Trusts. However, a previous study found that shift patterns tend to vary more within hospitals than between hospitals in England.[4]

For peer review only

For peer review only - http://bmjopen.bmj.com/site/about/guidelines.xhtml

CONCLUSIONS

This is the first study in nursing to use objective measures of shift patterns and missed care obtained from records spanning a long period of time. Where working hours involved a larger proportion of long shifts delays in obtaining scheduled vital signs observations by nursing support staff were observed. While this study lends partial support to previous findings using global self-report measures, further research should explore better measures of job performance and more complete data on costs, and effects, to work out which shift patterns are most cost-effective for ward-based services.

For peer review only

For peer review only - http://bmjopen.bmj.com/site/about/guidelines.xhtml

ACKNOWLEDGMENTS

The authors thank Portsmouth Hospitals NHS Trust for allowing us to use the study data.

FUNDING

This work was supported by NIHR Collaboration for Leadership in Applied Health Research and Care Wessex and by the NIHR HS&DR – grant number - 13/114/17: “Nurse staffing levels, missed vital signs observations and mortality in hospital wards: modelling the consequences and costs of variations in nurse staffing and skill mix”. The views expressed in this publication are those of the authors and not necessarily those of the NHS, the National Institute for Health Research or the Department of Health and Social Care.

CONFLICT OF INTEREST

The authors have no conflict of interest to declare.

AUTHORS CONTRIBUTIONS

CDO drafted the article, giving substantial contributions to its conception and design, collaborated in acquiring the data, performed statistical analyses and interpreted the data, and gave final approval of the version to be published. She agreed to be accountable for all aspects of the work in ensuring that questions related to the accuracy or integrity of any part of the work are appropriately investigated and resolved. PG gave substantial contributions to the conception and design of the work, the interpretation of data and revised critically the article for important intellectual content, and gave final approval of the version to be published. OR gave substantial contributions to acquiring the data, to the statistical analysis and data interpretation, and revised critically the article for important intellectual content, and gave final approval of the version to be published. AR-S collaborated in the conception of the study, revised critically the article for important intellectual content and gave final approval of the version to be published. PM gave substantial contributions to acquiring the data, collaborated with statistical analysis and data interpretation, revised critically the article for important intellectual content and gave final approval of the version to be published. JB gave substantial contributions to the conception of the study, revised critically the article for important intellectual content and gave final approval of the version to be published.

For peer review only - http://bmjopen.bmj.com/site/about/guidelines.xhtml

DATA SHARING AGREEMENT

Due to the nature of this study and the conditions attached to original data agreements, there is no data available for wider use. All queries should be submitted to the corresponding author in the first instance.

For peer review only

For peer review only - http://bmjopen.bmj.com/site/about/guidelines.xhtml

REFERENCES

1. NHS Evidence. Moving to 12-hour shift patterns: to increase continuity and reduce costs, 2010.

2. Dall'Ora C, Ball J, Recio-Saucedo A, et al. Characteristics of shift work and their impact on employee performance and wellbeing: A literature review. *Int J Nurs Stud* 2016;57:12-27. doi: 10.1016/j.ijnurstu.2016.01.007

3. Stimpfel AW, Aiken LH. Hospital staff nurses' shift length associated with safety and quality of care. *J Nurs Care Qual* 2013;28(2):122-9. doi: 10.1097/NCQ.0b013e3182725f09

4. Griffiths P, Dall'Ora C, Simon M, et al. Nurses' shift length and overtime working in 12 European countries: The association with perceived quality of care and patient safety. *Med Care* 2014;52(11):975-81. doi: 10.1097/MLR.0000000000000233

5. Rogers AE, Hwang WT, Scott LD, et al. The working hours of hospital staff nurses and patient safety. *Health Aff (Millwood)* 2004;23(4):202-12. doi: 10.1377/hlthaff.23.4.202 [published Online First: 2004/08/21]

6. Clendon J, Gibbons V. 12 h shifts and rates of error among nurses: A systematic review. *Int J Nurs Stud*

For peer review only

2015;52(7):1231-42. doi: 10.1016/j.ijnurstu.2015.03.011 [published Online First: 2015/04/26]

7. Trinkoff AM, Johantgen M, Storr CL, et al. Nurses' work schedule characteristics, nurse staffing, and patient mortality. *Nurs Res* 2011;60(1):1-8. doi: 10.1097/NNR.0b013e3181fff15d [published Online First: 2010/12/04]

8. Ball J, Day T, Murrells T, et al. Cross-sectional examination of the association between shift length and hospital nurses job satisfaction and nurse reported quality measures. *BMC Nurs* 2017;16(1):26. doi: 10.1186/s12912-017-0221-7

9. Reid N, Robinson G, Todd C. The quantity of nursing care on wards working 8- and 12-hour shifts. *Int J Nurs Stud* 1993;30(5):403-13. doi: https://doi.org/10.1016/0020-7489(93)90050-5

10. Antonakis J, Bendahan S, Jacquart P, et al. On making causal claims: A review and recommendations. *Leadership Quart* 2010;21(6):1086-120. doi: 10.1016/j.leaqua.2010.10.010

11. Folkard S, Tucker P. Shift work, safety and productivity. *Occup Med (Lond)* 2003;53(2):95-101. doi: 10.1093/occmed/kqg047

For peer review only - http://bmjopen.bmj.com/site/about/guidelines.xhtml

12. Griffiths P, Ball J, Drennan J, et al. Nurse staffing and patient outcomes: Strengths and limitations of the evidence to inform policy and practice. A review and discussion paper based on evidence reviewed for the National Institute for Health and Care Excellence Safe Staffing guideline development. *Int J Nurs Stud* 2016;63:213-25. doi: 10.1016/j.ijnurstu.2016.03.012 [published Online First: 2016/05/01]

13. Recio-Saucedo A, Dall'Ora C, Maruotti A, et al. What impact does nursing care left undone have on patient outcomes? Review of the literature. *J Clin Nurs* 2017;0(0) doi: doi:10.1111/jocn.14058

14. Smith GB. In-hospital cardiac arrest: is it time for an in-hospital 'chain of prevention'? *Resuscitation* 2010;81(9):1209-11. doi: 10.1016/j.resuscitation.2010.04.017 [published Online First: 2010/07/06]

15. Royal College of Physicians. National Early Warning (NEWS). Standardising the assessment of acute-illness severity in the NHS. London: Royal College of Physicians, 2012.

For peer review only - http://bmjopen.bmj.com/site/about/guidelines.xhtml

16. Smith GB, Prytherch DR, Meredith P, et al. The ability of the National Early Warning Score (NEWS) to discriminate patients at risk of early cardiac arrest, unanticipated intensive care unit admission, and death. *Resuscitation* 2013;84(4):465-70. doi: 10.1016/j.resuscitation.2012.12.016

17. Griffiths P, Recio-Saucedo A, Dall'Ora C, et al. The association between nurse staffing and omissions in nursing care: A systematic review. *J Adv Nurs* 2018;0(0) doi: doi:10.1111/jan.13564

18. R: A language and environment for statistical computing [program]. 1.1.442 version. Vienna, Austria: R Foundation for Statistical Computing, 2018.

19. lme4: Linear Mixed-Effects Models Using Eigen and S4 [program]. 1.1-13 version, 2015.

20. Barker LM, Nussbaum MA. Fatigue, performance and the work environment: a survey of registered nurses. *J Adv Nurs* 2011;67(6):1370-82. doi: 10.1111/j.1365-2648.2010.05597.x

21. Chen J, Davis KG, Daraiseh NM, et al. Fatigue and recovery in 12-hour dayshift hospital nurses. *J Nurs Manag* 2014;22(5):593-603. doi: 10.1111/jonm.12062

For peer review only - http://bmjopen.bmj.com/site/about/guidelines.xhtml

22. Bach S, Kessler I, Heron P. Nursing a Grievance? The Role of Healthcare Assistants in a Modernized National Health Service. *Gender, Work & Organization* 2012;19(2):205-24. doi: 10.1111/j.1468-0432.2009.00502.x

23. Hogan J. Why don't nurses monitor the respiratory rates of patients? *Br J Nurs* 2006;15(9):489-92. doi: 10.12968/bjon.2006.15.9.21087

24. Hope J, Recio-Saucedo A, Fogg C, et al. A fundamental conflict of care: Nurses' accounts of balancing patients' sleep with taking vital sign observations at night. *J Clin Nurs* 2017;0(0) doi: 10.1111/jocn.14234

25. Ball JE, Bruyneel L, Aiken LH, et al. Post-operative mortality, missed care and nurse staffing in nine countries: A cross-sectional study. *Int J Nurs Stud* 2018;78:10-15. doi: 10.1016/j.ijnurstu.2017.08.004

26. Griffiths P, Ball J, Bloor K, et al. Nurse staffing levels, missed vital signs observations and mortality in hospital wards: retrospective longitudinal observational study using routinely collected data - Final report. *Health Services and Delivery Research* 2018

For peer review only - http://bmjopen.bmj.com/site/about/guidelines.xhtml

Appendix 1 – HPPD, number of nursing staff and number of beds by ward

Ward	Number of beds	Number of Nursing Staff	HPPD Mean (SD)	Skill Mix* Mean (SD)
Oncology	40	109	7.2 (0.8)	0.71 (0.02)
Medical – Gastro	36	72	6.4 (1)	0.50 (0.07)
Coronary Care	23	48	7.2 (0.8)	0.83 (0.06)
Cardiology	36	62	5.6 (0.7)	0.51 (0.05)
Trauma 1	28	59	7.3 (1.1)	0.54 (0.06)
General Medicine 1	30	47	6.5 (1)	0.45 (0.05)
General Medicine 2	34	58	5.5 (0.8)	0.58 (0.05)
Trauma 2	26	55	6.8 (0.8)	0.55 (0.05)
Orthopaedics 1	36	46	6.4 (1.2)	0.56 (0.05)
Orthopaedics 2	36	62	7.4 (1.2)	0.52 (0.05)
Orthopaedics 3	13	31	8.1 (1.3)	0.56 (0.06)
Surgical 1	30	56	5.2 (0.5)	0.60 (0.05)
Surgical 2	32	61	5.4 (0.7)	0.59 (0.05)
Respiratory 1	36	60	5.4 (0.8)	0.50 (0.07)
Respiratory 2	40	83	7.1 (0.6)	0.67 (0.04)
Medicine for older people 1	13	79	7.7 (1.4)	0.50 (0.06)
Medicine for older people 2	30	25	7.3 (0.9)	0.46 (0.05)
Medicine for older people 3	23	73	6.5 (0.6)	0.52 (0.04)
Medicine for older people 4	29	84	6.9 (0.8)	0.52 (0.05)
Medicine for older people 5	30	49	7.6 (0.9)	0.47 (0.08)
Stroke Unit 1	25	62	7 (0.5)	0.46 (0.05)
Stroke Unit 2	34	85	6.7 (0.8)	0.53 (0.06)
Renal 1	10	47	11.2 (1.6)	0.78 (0.06)
Renal 2	26	56	8.4 (1)	0.79 (0.05)
Renal 3	14	31	8.6 (1.2)	0.75 (0.05)
Gynaecology	22	46	7.9 (1.5)	0.66 (0.07)
Head & Neck	27	51	6.3 (1.2)	0.68 (0.07)
Medical Assessment Unit	58	155	8.9 (1.3)	0.71 (0.03)
Private Patients Unit	13	30	8.3 (1.8)	0.69 (0.05)
Surgical Assessment Unit	28	53	5.2 (0.9)	0.65 (0.06)
Surgical High Intensity Unit	10	31	10 (1.5)	0.79 (0.06)
Urology	31	78	5.6 (0.7)	0.55 (0.04)

* Defined as RN-HPPD/(RN-HPPD + HCA-HPPD)

For peer review only

STROBE 2007 (v4) Statement—Checklist of items that should be included in reports of *cross-sectional studies*

Section/Topic	Item #	Recommendation	Reported on page #
**Title and abstract**	1	(*a*) Indicate the study's design with a commonly used term in the title or the abstract	1
		(*b*) Provide in the abstract an informative and balanced summary of what was done and what was found	3
**Introduction**			
Background/rationale	2	Explain the scientific background and rationale for the investigation being reported	5
Objectives	3	State specific objectives, including any prespecified hypotheses	6
**Methods**			
Study design	4	Present key elements of study design early in the paper	6
Setting	5	Describe the setting, locations, and relevant dates, including periods of recruitment, exposure, follow-up, and data collection	6-7
Participants	6	(*a*) Give the eligibility criteria, and the sources and methods of selection of participants	6-7
Variables	7	Clearly define all outcomes, exposures, predictors, potential confounders, and effect modifiers. Give diagnostic criteria, if applicable	6-7
Data sources/ measurement	8*	For each variable of interest, give sources of data and details of methods of assessment (measurement). Describe comparability of assessment methods if there is more than one group	6-7
Bias	9	Describe any efforts to address potential sources of bias	6-7
Study size	10	Explain how the study size was arrived at	6-7
Quantitative variables	11	Explain how quantitative variables were handled in the analyses. If applicable, describe which groupings were chosen and why	6-7
Statistical methods	12	(*a*) Describe all statistical methods, including those used to control for confounding	7
		(*b*) Describe any methods used to examine subgroups and interactions	N/A
		© Explain how missing data were addressed	N/A
		(*d*) If applicable, describe analytical methods taking account of sampling strategy	N/A
		€ Describe any sensitivity analyses	N/A
**Results**			

For peer review only

Participants	13*	(a) Report numbers of individuals at each stage of study—eg numbers potentially eligible, examined for eligibility, confirmed eligible, included in the study, completing follow-up, and analysed	8
		(b) Give reasons for non-participation at each stage	N/A
		© Consider use of a flow diagram	N/A
Descriptive data	14*	(a) Give characteristics of study participants (eg demographic, clinical, social) and information on exposures and potential confounders	8
		(b) Indicate number of participants with missing data for each variable of interest	N/A
Outcome data	15*	Report numbers of outcome events or summary measures	8
Main results	16	(*a*) Give unadjusted estimates and, if applicable, confounder-adjusted estimates and their precision (eg, 95% confidence interval). Make clear which confounders were adjusted for and why they were included	9
		(*b*) Report category boundaries when continuous variables were categorized	N/A
		© If relevant, consider translating estimates of relative risk into absolute risk for a meaningful time period	N/A
Other analyses	17	Report other analyses done—eg analyses of subgroups and interactions, and sensitivity analyses	N/A
**Discussion**			
Key results	18	Summarise key results with reference to study objectives	9
Limitations	19	Discuss limitations of the study, taking into account sources of potential bias or imprecision. Discuss both direction and magnitude of any potential bias	10-11
Interpretation	20	Give a cautious overall interpretation of results considering objectives, limitations, multiplicity of analyses, results from similar studies, and other relevant evidence	10-11
Generalisability	21	Discuss the generalisability (external validity) of the study results	11
**Other information**			
Funding	22	Give the source of funding and the role of the funders for the present study and, if applicable, for the original study on which the present article is based	3

*Give information separately for cases and controls in case-control studies and, if applicable, for exposed and unexposed groups in cohort and cross-sectional studies.

Note: An Explanation and Elaboration article discusses each checklist item and gives methodological background and published examples of transparent reporting. The STROBE checklist is best used in conjunction with this article (freely available on the Web sites of PLoS Medicine at http://www.plosmedicine.org/, Annals of Internal Medicine at http://www.annals.org/, and Epidemiology at http://www.epidem.com/). Information on the STROBE Initiative is available at www.strobe-statement.org.

For peer review only

BMJ Open

Nurses’ 12-hour shifts and missed or delayed vital signs observations on hospital wards: retrospective observational study

Journal:	*BMJ Open*
Manuscript ID	bmjopen-2018-024778.R2
Article Type:	Research
Date Submitted by the Author:	29-Oct-2018

Complete List of Authors:	Dall'Ora, Chiara; University of Southampton, Faculty of Health Sciences; NIHR Collaboration for Leadership in Applied Health Research and Care Wessex,<br>Griffiths, Peter; University of Southampton, Faculty of Health Sciences<br>Redfern, Oliver; University of Portsmouth , School of Computing; University of Oxford, Nuffield Department of Clinical Neurosciences<br>Recio-Saucedo, Alejandra; University of Southampton, Faculty of Health Sciences<br>Meredith, Paul; Portsmouth Hospitals NHS Trust, Research & Innovation Department<br>Ball, Jane; National Institute for Health Research Collaboration for Leadership in Applied Health Research and Care (Wessex),University of Southampton, Centre for Innovation and Leadership in Health Sciences

**Primary Subject Heading**:	Health services research
Secondary Subject Heading:	Nursing
Keywords:	Shift Work Schedule, Nursing, 12-hour Shifts, Vital Signs Monitoring, Missed Care

SCHOLARONE™
Manuscripts

TITLE PAGE

Nurses' 12-hour shifts and missed or delayed vital signs observations on hospital wards: retrospective observational study

Chiara Dall'Ora* [1,2] RN, MSc, PhD, University of Southampton, Building 67, Highfield, Southampton, SO17 1BJ, UK, C.Dall'ora@soton.ac.uk, +44(023) 8059 8249

Peter Griffiths*[1,2], Oliver Redfern[3,4], Alejandra Recio-Saucedo[2], Paul Meredith[5], Jane Ball[2], On behalf of the Missed Care Study Group†

* Chiara Dall'Ora and Peter Griffiths contributed equally to this manuscript

[1] National Institute for Health Research Collaboration for Leadership in Applied Health Research and Care (Wessex), University of Southampton, Southampton, UK

[2] Faculty of Health Sciences, University of Southampton, Southampton, UK

[3] School of Computing, University of Portsmouth, UK

[4] Kadoorie Centre for Critical Care Research and Education, Nuffield Department of Clinical Neurosciences, University of Oxford, UK

[5] Research & Innovation Department, Portsmouth Hospitals NHS Trust, Portsmouth, UK

† The Missed Care Study Group comprises Karen Bloor, University of York; Dankmar Böhning, University of Southampton; Jim Briggs, University of Portsmouth, Centre for Healthcare Modelling and Informatics; Anya De Iongh, Independent lay researcher; Jeremy Jones, University of Southampton, Faculty of Health Sciences; Caroline Kovacs, University of Portsmouth, Centre for Healthcare Modelling and Informatics; David Prytherch University of Portsmouth, Centre for Healthcare Modelling and Informatics and Portsmouth Hospitals NHS Trust, Clinical Outcomes Research Group; Paul Schmidt, National Institute for Health Research

For peer review only - http://bmjopen.bmj.com/site/about/guidelines.xhtml

For peer review only

Collaboration for Applied Health Research and Care (Wessex) and Portsmouth Hospitals NHS Trust, Clinical Outcomes Research Group; Nicky Sinden, Portsmouth Hospitals NHS Trust, Clinical Outcomes Research Group; Gary Smith, Bournemouth University

Word count: 2755

For peer review only

For peer review only - http://bmjopen.bmj.com/site/about/guidelines.xhtml

ABSTRACT

Objectives: 12-hour shifts worked by nurses on acute hospital wards have been associated with increased rates of missed care reported by nurses. This study aimed to measure the association between nurses working shifts of at least 12 hours and an objective measure of missed care – vital signs observations taken on time according to an acuity-based surveillance protocol.

Design: A retrospective observational study using routinely collected data from March 2012 to March 2015.

Setting: 32 general inpatient wards at a large acute hospital in England.

Participants: 658,628 nursing shifts nested in 24,069 ward-days.

Outcome measures: The rate of daily delayed and missed vital signs observations. We focussed on situations where vital signs observations were required at least every 4 hours and measured the number of instances where observations were delayed or missed, per 24-hour period. For each ward and each day, shift patterns were characterised in terms of proportion of care hours per patient day deriving from "long" shifts (≥12-hour) for both registered nurses and healthcare assistants.

Results: On 99,043 occasions (53%) observations were significantly delayed, and on 81,568 occasions (44%) observations were missed. Observations were more likely to be delayed when a higher proportion of the hours worked by healthcare assistants were part of long shifts (IRR = 1.05; 95% CI: 1.00-1.10). No significant association was found in relation to the proportion of hours registered nurses worked as long shifts.

Conclusion: On days when a higher proportion of hours worked by health care assistants are from long shifts, the risk of delaying vital signs observations is higher, suggesting lower job performance. While longer shifts are thought to require fewer staff resources to maintain nurse to patient ratios, any benefits may be lost if staff become less productive.

Keywords: Shift Work Schedule; Nursing; 12-hour shifts; Vital Signs Monitoring; Missed Care

For peer review only - http://bmjopen.bmj.com/site/about/guidelines.xhtml

STRENGTHS AND LIMITATIONS OF THIS STUDY

- This study was able to measure an aspect of missed care through an objective outcome, delayed / missed vital signs observations – as opposed to nurse self-reports
- This study drew on a large dataset of nurses' shifts and patients' vital signs observations and covered three years (2012 – 2015)
- However, the study was carried out in a single hospital
- Due to the nature of the administrative datasets available, we were not able to control for staff characteristics including age

For peer review only

BACKGROUND

Shifts of 12 hours or longer are becoming increasingly common in nursing, based on perceived efficiency savings achieved by reducing the overlap between shorter shifts associated with a three shift system.[1] The same average patients-to-nurse ratio could potentially be kept with a lower number of nursing hours, although claims of improved efficiency are yet to be verified in empirical studies. Furthermore, there is no evidence to suggest that organising the delivery of nursing services in acute hospitals with ≥12-hour ("long") shifts improves the safety or efficacy of patient care. A recent literature review found no evidence of a beneficial effect of long shifts on patient outcomes,[2] with findings from a number of studies indicating that greater use of long shifts in hospital wards is associated with a number of adverse outcomes, including reducing the quality of care and patient safety;[3 4] higher rates of errors;[5 6] and increased mortality.[7]

Recent studies have examined the impact of long shifts on nurses' ability to complete all patient care on a given shift. These studies drew on data from 12 European countries and found that nurses working 12-hour shifts were more likely to report higher levels of care being left undone due to lack of time, a phenomenon generally referred to as "missed care".[8] The underlying mechanism for this association has not been studied, although it has been hypothesised that nurses may need to pace during long shifts in order to maintain enough energy until the end of the shift.[9] This would suggest that nurses' productivity when working 12-hour shifts may be lower, potentially counteracting the expected efficiency savings.

Studies examining the association between long shifts and missed care have relied exclusively on nurse-reported measures, which can result in common-methods bias.[10] Although the direct study of worker performance under different shift systems' is acknowledged to be challenging,[11] recent expansion in hospital information systems have created new opportunities to study the timely completion of nursing work.[12]

There is now substantial evidence that missed care impacts negatively on patients, with consequences ranging from poor experience of care to increased risk of infection, readmissions and adverse outcomes from undetected physiological deterioration.[13] Timely monitoring and recording of patients' vital signs is key to identify patient deterioration[14] and electronic recording of these observations provides an opportunity to examine an objective indicator of nurses' job performance, which has not been previously explored.

For peer review only - http://bmjopen.bmj.com/site/about/guidelines.xhtml

Using routinely collected data, the aim of this study was to investigate the association between the length of shifts worked by nursing staff and adherence to a vital signs observations protocol.

METHODS

This was a retrospective observational study using routinely collected data on nursing staff, shift patterns and patient vital signs observations. Data were extracted for all general adult wards (n= 32) in an acute care hospital Trust in the South of England over a three-year period (1 April 2012- 31 March 2015). These data were obtained as part of a parent study (ISRCTN registration: 17930973 http://www.isrctn.com/ISRCTN17930973). Ethical approval to undertake this research was granted from the University of Southampton Ethics Committee (Submission Number 18311).

Data Sources and variables

Records of worked nursing shifts were extracted from an electronic rostering system implemented hospital-wide and directly linked to payroll. Patient data were extracted from the Patient Administration Systems and were linked to the roster data at the ward level. The patient sample included all patients who were on the wards in the days included in the analysis. All data were pseudo-anonymised at source before being provided to the research team.

The nursing workforce is composed of registered nurses (RNs) and unregistered health care assistants (HCAs), also known as healthcare support workers, or “nursing support staff”. For each ward for each day, we calculated registered nurses hours per patient day (RN-HPPD) and healthcare assistants hours per patient day (HCA-HPPD). In each case this was calculated as staff hours / (patient hours/24); a patient day is equivalent to one patient occupying one bed for a full 24 hours. The proportion of care hours per patient day derived from ≥12-hour shifts was calculated for each day in each ward.

Vital signs observations were derived from a database of records made using the VitalPac™ system, where nurses record clinical data on hand held devices at the bedside. These systems comprise the only official record of observations that are maintained on the wards in the study and the use of the system is mandated by Trust policy. The frequency of vital signs observations required by a patient at any given time is determined by a protocol based on the National Early Warning Score (NEWS), which defines when the next observation is due.

For peer review only - http://bmjopen.bmj.com/site/about/guidelines.xhtml

Detailed description of NEWS can be found elsewhere;[15] in broad terms, it requires that acutely ill patients are observed more often and defines a minimum observation frequency for a given level of patient acuity.

For each set of observations, we obtained the expected time to the next observation (based on the protocol) and classified the subsequent observation as delayed (after 1/3 of the defined interval had elapsed) or missed (after 2/3 of the defined interval had elapsed). This classification was arrived at after extensive discussion, examination of the distribution of delays in taking observations and consultation with patient and professional stakeholders, including staff nurses, senior nurses, physicians, and clinical data analysists. The classification of missed observations reflects that an observation taken late, but near to the time a subsequent observation is due, is more clearly fulfilling the second scheduled observation than the first.

According to the hospital escalation protocol, patients are considered at high risk when their NEWS score is six or above, requiring observations at least once every four hours. Since patients with high NEWS scores are those more likely to die and/or to experience critical deterioration[16] we considered only observations of patients who exhibited NEWS ≥ 6. This is the group who are deemed to most require the observations undertaking, and whose outcomes are most vulnerable to deterioration if not sufficiently monitored.

Statistical analysis

The first stage of data analysis involved a descriptive analysis of frequencies and summaries of shift patterns and delayed and missed vital signs observations. The Pearson correlation between the proportion of HPPD deriving from ≥12-hour shifts and missed, and delayed observations was calculated for both registered nurses and healthcare assistants.

Linear mixed-effects Poisson models were then fit. These models sought to examine the effect of higher proportions of registered nurse hours per patient day (RN-HPPD) and healthcare assistant hours per patient day (HCA-HPPD) derived from ≥12-hour shifts on the likelihood of delaying or missing a vital signs observation. Analyses were performed at the ward-day level and delayed/missed vital signs observations were treated as count outcome; the number of observations due was used as an offset. Registered nurse hours per patient day (RN-HPPD) and healthcare assistant hours per patient day (HCA-HPPD) were added as control variables. Intraclass correlation coefficients (ICCs) were computed from unconditional random intercept models to describe the between-ward variation of delayed and missed vital signs observations. The ward level ICC for both delayed and missed observations was 0.37. Due to the high ICC

For peer review only - http://bmjopen.bmj.com/site/about/guidelines.xhtml

and the data structure (i.e. observations aggregated at the ward-day level), ward was included as a random effect in the multivariate models. As missed care is associated with total staffing levels,[17] we included both RN and HCA-HPPD in all models as control variables. All analysis was undertaken in the R statistical environment version 3.3.2[18] and mixed effects models were fit using the lme4 package.[19]

Patient involvement

The parent project was developed with significant input from local public and patient groups who helped shape the original research focus and with a co-researcher who was actively involved in day-to-day project activities including discussion and interpretation of results. As part of the parent study, we undertook a series of consultations with public, patient and clinical experts in order to identify issues that should be considered, including determining an acceptable level of vital signs observations compliance (completing a full set of observations in time). The work was completed in stages so that different groups were approached separately to share views relevant to each individual group. Our approach, co-designed with the parent's study Patient and Public Involvement (PPI) representative / lay co-researcher was intended to make the key issues to be discussed tangible for a more lay audience and professional stakeholders such as staff nurses.

RESULTS

There were 1944 nursing staff members in the sample. 1244 staff members were registered nurses and 700 staff members were classified as healthcare assistants. Nursing staff worked on average 276 shifts within the three-year study period. In total, 390,325 shifts were worked by RNs and 268,303 shifts were worked by HCAs. The patient population comprised of 142,046 patients; their mean age was 67; the majority (79%) were emergency admissions, and 50% of the patients had no comorbidities (Charlson comorbidity index = 0). The total number of observations in high acuity patients was 184,628. The total number of delayed observations in high acuity patients was 99,043 (53%), while the missed observations were 81,568 (44%), nested in 24,069 ward-days. Wards differed as regards size (i.e. number of beds), mean HPPD and mean skill mix (i.e. the proportion of RN-HPPD over the total HPPD, calculated as RN-HPPD/(RN-HPPD + HCA-HPPD)). For a detailed description of ward characteristics, please see Appendix 1.

For peer review only - http://bmjopen.bmj.com/site/about/guidelines.xhtml

Table 1 summarises the distribution of expected, delayed and missed vital signs observations; and of the proportion of HCA and RN-HPPD derived from ≥12-hour shifts.

Table 1 Distribution of missed and delayed observations; and proportion of HPPD deriving from long shifts

	Mean	Median	Interquartile range (IQR)	Min	Max
Expected observations per 24 hours in each ward	7.6	5	2-10	1	87
Delayed observations per 24 hours in each ward	4.1	2	1 – 5	0	56
Missed observations per 24 hours in each ward	3.4	2	1 – 4	0	51
Percentage of HCA-HPPD derived by ≥12-h shifts	46%	57%	0% – 79%	0%	100%
Percentage of RN-HPPD derived by ≥12-h shifts	48%	61%	0% – 82%	0%	100%

The mean number of delayed observations in each ward-day was 4.1, while on average 3.4 observations were missed on each ward-day. Across the three years, on average 46% of HCA-HPPD derived from ≥12-hour shifts, with large variation (Interquartile range (IQR) 0% –79%) and on average 48% of RN-HPPD derived from ≥12-hour shifts (IQR 0% –82%).

The proportion of RN-HPPD contributed by ≥12-hour shifts was positively correlated with the rate of both delayed (r = 0.26) and missed observations (r = 0.25). Similarly, the proportion of HCA-HPPD deriving from ≥12-hour shifts was also correlated with delayed (r = 0.27) and missed observations (r = 0.27). Table 2 reports the association between the proportion of RN-HCPPD and HCA-HCPPD deriving from ≥ 12-hour shifts and delayed and missed vital signs observations.

For peer review only - http://bmjopen.bmj.com/site/about/guidelines.xhtml

Table 2 Multivariable association between the proportions of RN-HPPD, HCA-HPPD deriving from 12 hours or more shifts and delayed/missed vital signs observations

**Delayed vital signs observations**		
**Shift characteristics**	**IRR†**	**95% CI**
Proportion of RN-HPPD deriving from ≥12-h shifts	0.96	0.91-1.01
Proportion of HCA-HPPD deriving from ≥12-h shifts	1.05*	1.00-1.10±
**Missed vital signs observations**		
**Shift characteristics**	**IRR**	**95% CI**
Proportion of RN-HPPD deriving from ≥12-h shifts	0.96	0.91-1.01
Proportion of HCA-HPPD deriving from ≥12-h shifts	1.04	0.99-1.09

* Statistically significant at $p< 0.05$

† Incidence Rate Ratio (all models include random effect for ward and RN / HCA HPPD)
Random effect: ward

± The lower bound 95% confidence interval for proportion of HCA-HPPD deriving from ≥12-h shifts is 1.0004, and it has been rounded to 1.00 for reporting purposes

In multivariate models controlling for staffing levels, the proportion of HCA-HPPD derived by ≥12-hour shifts was significantly associated with rate of delayed vital signs observations. An additional 1% of HCA-HPPD derived from long shifts was associated with a 5% increase in the rate of delayed observations compared to days where no long shifts were worked by HCAs (IRR = 1.05; 95% CI: 1.00-1.10). Results for missed observations were similar but not statistically significant. When looking at registered nurses, the proportion of hours worked through long shifts by RNs was not significantly associated with neither delayed nor missed vital signs observations.

To exclude the possibility that the use of ≥12-hour shifts was associated with increased workload of more acute patients, we ran a linear mixed model to establish how proportions of acutely ill patients (NEWS ≥6) on a ward-day were associated with proportions of RN-HPPD

For peer review only - http://bmjopen.bmj.com/site/about/guidelines.xhtml

and HCA-HPPD deriving from 12-hour shifts, and we found no association (results available from authors).

DISCUSSION

This is the first study to explore the relationship between long nursing shifts and an objective measure of missed care in terms of missed/delayed vital signs observations. We found that delays in obtaining vital signs observations were relatively common for patients assessed as requiring frequent observation. For HCAs, where more of their hours were worked as long shifts, there was a significant increase in delayed vital signs observations. We did not find a significant relationship between delayed/missed vital signs observations and the proportion of RN HPPD worked as part of long shifts.

In the context of a growing body of research suggesting that long nursing shifts on hospital wards are associated with lower quality of care and worse patient outcomes, this finding points to one possible mechanism, as using vital signs observations as an indicator, job performance would appear to be lower for HCAs working longer shifts. This in turn is likely to be a product of the increased fatigue that is frequently reported by those working long shifts.[20 21]

Previous research suggesting that the job performance of nursing staff may reduce when working 12-hour shifts has focussed specifically on RNs,[4 9 20] but we found no significant association for this staff group. These studies have relied on general reports of missed care whereas we focussed specifically on vital signs observations. Evidence of the association between staffing levels and missed care shows that, in general, RNs are more likely to report that lack of time causes them to miss interpersonal care (e.g. talking and comforting patients and planning patient care) than "clinical" care.[17] This may reflect a prioritisation of some aspects of care by RNs in the face of limited capacity. It might be that other aspects of RN work are adversely affected by 12-hour shifts, but observation of patient groups identified as at risk of deterioration remains a priority.

The monitoring of vital signs is increasingly being delegated to HCAs. Research undertaken with registered nurses and healthcare assistants in the UK found that vital signs observations were a central feature of healthcare assistants' roles.[22] Furthermore, there are reports of registered nurses considering the assessment of vital signs a basic task and therefore one that RNs may delegate to less qualified staff members.[23] The extent to which such delegation has occurred in the present Trust is unclear, as the record of who undertook the observation was

For peer review only - http://bmjopen.bmj.com/site/about/guidelines.xhtml

incomplete and unreliable, although all health care assistants are expected to have the competency to undertake and record vital signs observations. Being unable to establish whether such delegation occurs prevents us from understanding the role HCAs may play in avoiding nursing missed care. Anecdotal accounts suggest that HCAs undertaking vital signs is widespread. Thus, it may simply be that compliance with the vital signs observation protocol is a good indicator of job performance for HCAs but not for RNs because of different responsibilities. Alternatively, it may be that long shifts adversely affect HCAs but not RNs or that RNs place a higher priority on ensuring timely observation even though their overall capacity is similarly affected by long shifts.

This study has some limitations. We were only able to focus on a single aspect of job performance - vital signs monitoring - which may not be reflective of global performance. We were also unable to ascertain which staff group was assigned to take observations. Furthermore, it was not possible to determine whether observations were deliberately omitted for clinical reasons despite being in contravention of the protocol. For example, in a previous study nursing staff have reported choosing not to perform some observations at night because they felt waking patients could have a negative effect on individual patients. These reasons included patients who had difficulty sleeping and 'confused' patients who may become agitated.[24] Furthermore, patients may not have been available at the time observations were due, for example if they were undergoing diagnostic tests (e.g. radiology) elsewhere in the hospital. We did not study the direct consequences of missed vital signs observations, although other studies have shown that missed care in general is associated with adverse patient outcomes.[13] Recent studies have shown that nurse-reported missed care mediates the relationship between low staffing and mortality,[25] with a further study showing specifically that missed vital signs observations mediated an association between RN staffing levels and mortality.[26]

We were not able to account for staff characteristics including age and personal commitments external to work, both of which may influence job performance. Furthermore, in common with several previous missed care studies,[17] we weren't able to obtain a nurse-reported reason for missing vital signs observations. This study was conducted in a single site, and these findings may not generalise to other NHS Trusts. However, a previous study found that shift patterns tend to vary more within hospitals than between hospitals in England.[4]

For peer review only - http://bmjopen.bmj.com/site/about/guidelines.xhtml

CONCLUSIONS

This is the first study in nursing to use objective measures of shift patterns and missed care obtained from records spanning a long period of time. Where working hours involved a larger proportion of long shifts delays in obtaining scheduled vital signs observations by nursing support staff were observed. While this study lends partial support to previous findings using global self-report measures, further research should explore better measures of job performance and more complete data on costs, and effects, to work out which shift patterns are most cost-effective for ward-based services.

For peer review only

For peer review only - http://bmjopen.bmj.com/site/about/guidelines.xhtml

ACKNOWLEDGMENTS

The authors thank Portsmouth Hospitals NHS Trust for allowing us to use the study data.

FUNDING

This work was supported by NIHR Collaboration for Leadership in Applied Health Research and Care Wessex and by the NIHR HS&DR – grant number - 13/114/17: "Nurse staffing levels, missed vital signs observations and mortality in hospital wards: modelling the consequences and costs of variations in nurse staffing and skill mix". The views expressed in this publication are those of the authors and not necessarily those of the NHS, the National Institute for Health Research or the Department of Health and Social Care.

CONFLICT OF INTEREST

The authors have no conflict of interest to declare.

AUTHORS CONTRIBUTIONS

CDO drafted the article, giving substantial contributions to its conception and design, collaborated in acquiring the data, performed statistical analyses and interpreted the data, and gave final approval of the version to be published. She agreed to be accountable for all aspects of the work in ensuring that questions related to the accuracy or integrity of any part of the work are appropriately investigated and resolved. PG gave substantial contributions to the conception and design of the work, the interpretation of data and revised critically the article for important intellectual content, and gave final approval of the version to be published. OR gave substantial contributions to acquiring the data, to the statistical analysis and data interpretation, and revised critically the article for important intellectual content, and gave final approval of the version to be published. AR-S collaborated in the conception of the study, revised critically the article for important intellectual content and gave final approval of the version to be published. PM gave substantial contributions to acquiring the data, collaborated with statistical analysis and data interpretation, revised critically the article for important intellectual content and gave final approval of the version to be published. JB gave substantial contributions to the conception of the study, revised critically the article for important intellectual content and gave final approval of the version to be published.

For peer review only - http://bmjopen.bmj.com/site/about/guidelines.xhtml

DATA SHARING AGREEMENT

Due to the nature of this study and the conditions attached to original data agreements, there is no data available for wider use. All queries should be submitted to the corresponding author in the first instance.

For peer review only

For peer review only - http://bmjopen.bmj.com/site/about/guidelines.xhtml

REFERENCES

1. NHS Evidence. Moving to 12-hour shift patterns: to increase continuity and reduce costs, 2010.

2. Dall'Ora C, Ball J, Recio-Saucedo A, et al. Characteristics of shift work and their impact on employee performance and wellbeing: A literature review. *Int J Nurs Stud* 2016;57:12-27. doi: 10.1016/j.ijnurstu.2016.01.007

3. Stimpfel AW, Aiken LH. Hospital staff nurses' shift length associated with safety and quality of care. *J Nurs Care Qual* 2013;28(2):122-9. doi: 10.1097/NCQ.0b013e3182725f09

4. Griffiths P, Dall'Ora C, Simon M, et al. Nurses' shift length and overtime working in 12 European countries: The association with perceived quality of care and patient safety. *Med Care* 2014;52(11):975-81. doi: 10.1097/MLR.0000000000000233

5. Rogers AE, Hwang WT, Scott LD, et al. The working hours of hospital staff nurses and patient safety. *Health Aff (Millwood)* 2004;23(4):202-12. doi: 10.1377/hlthaff.23.4.202 [published Online First: 2004/08/21]

6. Clendon J, Gibbons V. 12 h shifts and rates of error among nurses: A systematic review. *Int J Nurs Stud*

For peer review only - http://bmjopen.bmj.com/site/about/guidelines.xhtml

2015;52(7):1231-42. doi: 10.1016/j.ijnurstu.2015.03.011 [published Online First: 2015/04/26]

7. Trinkoff AM, Johantgen M, Storr CL, et al. Nurses' work schedule characteristics, nurse staffing, and patient mortality. *Nurs Res* 2011;60(1):1-8. doi: 10.1097/NNR.0b013e3181fff15d [published Online First: 2010/12/04]

8. Ball J, Day T, Murrells T, et al. Cross-sectional examination of the association between shift length and hospital nurses job satisfaction and nurse reported quality measures. *BMC Nurs* 2017;16(1):26. doi: 10.1186/s12912-017-0221-7

9. Reid N, Robinson G, Todd C. The quantity of nursing care on wards working 8- and 12-hour shifts. *Int J Nurs Stud* 1993;30(5):403-13. doi: https://doi.org/10.1016/0020-7489(93)90050-5

10. Antonakis J, Bendahan S, Jacquart P, et al. On making causal claims: A review and recommendations. *Leadership Quart* 2010;21(6):1086-120. doi: 10.1016/j.leaqua.2010.10.010

11. Folkard S, Tucker P. Shift work, safety and productivity. *Occup Med (Lond)* 2003;53(2):95-101. doi: 10.1093/occmed/kqg047

For peer review only - http://bmjopen.bmj.com/site/about/guidelines.xhtml

12. Griffiths P, Ball J, Drennan J, et al. Nurse staffing and patient outcomes: Strengths and limitations of the evidence to inform policy and practice. A review and discussion paper based on evidence reviewed for the National Institute for Health and Care Excellence Safe Staffing guideline development. *Int J Nurs Stud* 2016;63:213-25. doi: 10.1016/j.ijnurstu.2016.03.012 [published Online First: 2016/05/01]

13. Recio-Saucedo A, Dall'Ora C, Maruotti A, et al. What impact does nursing care left undone have on patient outcomes? Review of the literature. *J Clin Nurs* 2017;0(0) doi: doi:10.1111/jocn.14058

14. Smith GB. In-hospital cardiac arrest: is it time for an in-hospital 'chain of prevention'? *Resuscitation* 2010;81(9):1209-11. doi: 10.1016/j.resuscitation.2010.04.017 [published Online First: 2010/07/06]

15. Royal College of Physicians. National Early Warning (NEWS). Standardising the assessment of acute-illness severity in the NHS. London: Royal College of Physicians, 2012.

For peer review only - http://bmjopen.bmj.com/site/about/guidelines.xhtml

16. Smith GB, Prytherch DR, Meredith P, et al. The ability of the National Early Warning Score (NEWS) to discriminate patients at risk of early cardiac arrest, unanticipated intensive care unit admission, and death. *Resuscitation* 2013;84(4):465-70. doi: 10.1016/j.resuscitation.2012.12.016

17. Griffiths P, Recio-Saucedo A, Dall'Ora C, et al. The association between nurse staffing and omissions in nursing care: A systematic review. *J Adv Nurs* 2018;0(0) doi: doi:10.1111/jan.13564

18. R: A language and environment for statistical computing [program]. 1.1.442 version. Vienna, Austria: R Foundation for Statistical Computing, 2018.

19. lme4: Linear Mixed-Effects Models Using Eigen and S4 [program]. 1.1-13 version, 2015.

20. Barker LM, Nussbaum MA. Fatigue, performance and the work environment: a survey of registered nurses. *J Adv Nurs* 2011;67(6):1370-82. doi: 10.1111/j.1365-2648.2010.05597.x

21. Chen J, Davis KG, Daraiseh NM, et al. Fatigue and recovery in 12-hour dayshift hospital nurses. *J Nurs Manag* 2014;22(5):593-603. doi: 10.1111/jonm.12062

For peer review only

For peer review only - http://bmjopen.bmj.com/site/about/guidelines.xhtml

22. Bach S, Kessler I, Heron P. Nursing a Grievance? The Role of Healthcare Assistants in a Modernized National Health Service. *Gender, Work & Organization* 2012;19(2):205-24. doi: 10.1111/j.1468-0432.2009.00502.x

23. Hogan J. Why don't nurses monitor the respiratory rates of patients? *Br J Nurs* 2006;15(9):489-92. doi: 10.12968/bjon.2006.15.9.21087

24. Hope J, Recio-Saucedo A, Fogg C, et al. A fundamental conflict of care: Nurses' accounts of balancing patients' sleep with taking vital sign observations at night. *J Clin Nurs* 2017;0(0) doi: 10.1111/jocn.14234

25. Ball JE, Bruyneel L, Aiken LH, et al. Post-operative mortality, missed care and nurse staffing in nine countries: A cross-sectional study. *Int J Nurs Stud* 2018;78:10-15. doi: 10.1016/j.ijnurstu.2017.08.004

26. Griffiths P, Ball J, Bloor K, et al. Nurse staffing levels, missed vital signs observations and mortality in hospital wards: retrospective longitudinal observational study using routinely collected data - Final report. *Health Services and Delivery Research* 2018

For peer review only

For peer review only - http://bmjopen.bmj.com/site/about/guidelines.xhtml

Appendix 1 – HPPD, number of nursing staff and number of beds by ward

Ward	Number of beds	Number of Nursing Staff	HPPD Mean (SD)	Skill Mix* Mean (SD)
Oncology	40	109	7.2 (0.8)	0.71 (0.02)
Medical – Gastro	36	72	6.4 (1)	0.50 (0.07)
Coronary Care	23	48	7.2 (0.8)	0.83 (0.06)
Cardiology	36	62	5.6 (0.7)	0.51 (0.05)
Trauma 1	28	59	7.3 (1.1)	0.54 (0.06)
General Medicine 1	30	47	6.5 (1)	0.45 (0.05)
General Medicine 2	34	58	5.5 (0.8)	0.58 (0.05)
Trauma 2	26	55	6.8 (0.8)	0.55 (0.05)
Orthopaedics 1	36	46	6.4 (1.2)	0.56 (0.05)
Orthopaedics 2	36	62	7.4 (1.2)	0.52 (0.05)
Orthopaedics 3	13	31	8.1 (1.3)	0.56 (0.06)
Surgical 1	30	56	5.2 (0.5)	0.60 (0.05)
Surgical 2	32	61	5.4 (0.7)	0.59 (0.05)
Respiratory 1	36	60	5.4 (0.8)	0.50 (0.07)
Respiratory 2	40	83	7.1 (0.6)	0.67 (0.04)
Medicine for older people 1	13	79	7.7 (1.4)	0.50 (0.06)
Medicine for older people 2	30	25	7.3 (0.9)	0.46 (0.05)
Medicine for older people 3	23	73	6.5 (0.6)	0.52 (0.04)
Medicine for older people 4	29	84	6.9 (0.8)	0.52 (0.05)
Medicine for older people 5	30	49	7.6 (0.9)	0.47 (0.08)
Stroke Unit 1	25	62	7 (0.5)	0.46 (0.05)
Stroke Unit 2	34	85	6.7 (0.8)	0.53 (0.06)
Renal 1	10	47	11.2 (1.6)	0.78 (0.06)
Renal 2	26	56	8.4 (1)	0.79 (0.05)
Renal 3	14	31	8.6 (1.2)	0.75 (0.05)
Gynaecology	22	46	7.9 (1.5)	0.66 (0.07)
Head & Neck	27	51	6.3 (1.2)	0.68 (0.07)
Medical Assessment Unit	58	155	8.9 (1.3)	0.71 (0.03)
Private Patients Unit	13	30	8.3 (1.8)	0.69 (0.05)
Surgical Assessment Unit	28	53	5.2 (0.9)	0.65 (0.06)
Surgical High Intensity Unit	10	31	10 (1.5)	0.79 (0.06)
Urology	31	78	5.6 (0.7)	0.55 (0.04)

* Defined as RN-HPPD/(RN-HPPD + HCA-HPPD)

For peer review only

STROBE 2007 (v4) Statement—Checklist of items that should be included in reports of *cross-sectional studies*

Section/Topic	Item #	Recommendation	Reported on page #
**Title and abstract**	1	(*a*) Indicate the study's design with a commonly used term in the title or the abstract	1
		(*b*) Provide in the abstract an informative and balanced summary of what was done and what was found	3
**Introduction**			
Background/rationale	2	Explain the scientific background and rationale for the investigation being reported	5
Objectives	3	State specific objectives, including any prespecified hypotheses	6
**Methods**			
Study design	4	Present key elements of study design early in the paper	6
Setting	5	Describe the setting, locations, and relevant dates, including periods of recruitment, exposure, follow-up, and data collection	6-7
Participants	6	(*a*) Give the eligibility criteria, and the sources and methods of selection of participants	6-7
Variables	7	Clearly define all outcomes, exposures, predictors, potential confounders, and effect modifiers. Give diagnostic criteria, if applicable	6-7
Data sources/ measurement	8*	For each variable of interest, give sources of data and details of methods of assessment (measurement). Describe comparability of assessment methods if there is more than one group	6-7
Bias	9	Describe any efforts to address potential sources of bias	6-7
Study size	10	Explain how the study size was arrived at	6-7
Quantitative variables	11	Explain how quantitative variables were handled in the analyses. If applicable, describe which groupings were chosen and why	6-7
Statistical methods	12	(*a*) Describe all statistical methods, including those used to control for confounding	7
		(*b*) Describe any methods used to examine subgroups and interactions	N/A
		© Explain how missing data were addressed	N/A
		(*d*) If applicable, describe analytical methods taking account of sampling strategy	N/A
		€ Describe any sensitivity analyses	N/A
**Results**			

For peer review only

Participants	13*	(a) Report numbers of individuals at each stage of study—eg numbers potentially eligible, examined for eligibility, confirmed eligible, included in the study, completing follow-up, and analysed	8
		(b) Give reasons for non-participation at each stage	N/A
		© Consider use of a flow diagram	N/A
Descriptive data	14*	(a) Give characteristics of study participants (eg demographic, clinical, social) and information on exposures and potential confounders	8
		(b) Indicate number of participants with missing data for each variable of interest	N/A
Outcome data	15*	Report numbers of outcome events or summary measures	8
Main results	16	(*a*) Give unadjusted estimates and, if applicable, confounder-adjusted estimates and their precision (eg, 95% confidence interval). Make clear which confounders were adjusted for and why they were included	9
		(*b*) Report category boundaries when continuous variables were categorized	N/A
		© If relevant, consider translating estimates of relative risk into absolute risk for a meaningful time period	N/A
Other analyses	17	Report other analyses done—eg analyses of subgroups and interactions, and sensitivity analyses	N/A
**Discussion**			
Key results	18	Summarise key results with reference to study objectives	9
Limitations	19	Discuss limitations of the study, taking into account sources of potential bias or imprecision. Discuss both direction and magnitude of any potential bias	10-11
Interpretation	20	Give a cautious overall interpretation of results considering objectives, limitations, multiplicity of analyses, results from similar studies, and other relevant evidence	10-11
Generalisability	21	Discuss the generalisability (external validity) of the study results	11
**Other information**			
Funding	22	Give the source of funding and the role of the funders for the present study and, if applicable, for the original study on which the present article is based	3

*Give information separately for cases and controls in case-control studies and, if applicable, for exposed and unexposed groups in cohort and cross-sectional studies.

Note: An Explanation and Elaboration article discusses each checklist item and gives methodological background and published examples of transparent reporting. The STROBE checklist is best used in conjunction with this article (freely available on the Web sites of PLoS Medicine at http://www.plosmedicine.org/, Annals of Internal Medicine at http://www.annals.org/, and Epidemiology at http://www.epidem.com/). Information on the STROBE Initiative is available at www.strobe-statement.org.

For peer review only
